# Single-cell transcriptomics captures features of human midbrain development and dopamine neuron diversity in brain organoids

**DOI:** 10.1038/s41467-021-27464-5

**Published:** 2021-12-15

**Authors:** Alessandro Fiorenzano, Edoardo Sozzi, Marcella Birtele, Janko Kajtez, Jessica Giacomoni, Fredrik Nilsson, Andreas Bruzelius, Yogita Sharma, Yu Zhang, Bengt Mattsson, Jenny Emnéus, Daniella Rylander Ottosson, Petter Storm, Malin Parmar

**Affiliations:** 1 Developmental and Regenerative Neurobiology, Wallenberg Neuroscience Center, and Lund Stem Cell Center, Department of Experimental Medical Science, Lund University, Lund, Sweden; 2 Regenerative Neurophysiology, Wallenberg Neuroscience Center, Lund Stem Cell Center, Department of Experimental Medical Science, Lund University, Lund, Sweden; 3 Department of Biotechnology and Biomedicine (DTU Bioengineering), Technical University of Denmark, Lyngby, Denmark

**Keywords:** Cellular neuroscience, RNA sequencing, Biomedical engineering, Pluripotent stem cells, Biomaterials - cells

## Abstract

Three-dimensional brain organoids have emerged as a valuable model system for studies of human brain development and pathology. Here we establish a midbrain organoid culture system to study the developmental trajectory from pluripotent stem cells to mature dopamine neurons. Using single cell RNA sequencing, we identify the presence of three molecularly distinct subtypes of human dopamine neurons with high similarity to those in developing and adult human midbrain. However, despite significant advancements in the field, the use of brain organoids can be limited by issues of reproducibility and incomplete maturation which was also observed in this study. We therefore designed bioengineered ventral midbrain organoids supported by recombinant spider-silk microfibers functionalized with full-length human laminin. We show that silk organoids reproduce key molecular aspects of dopamine neurogenesis and reduce inter-organoid variability in terms of cell type composition and dopamine neuron formation.

## Abstract (web summary)

3D brain organoids have been used to investigate human brain development and pathology. Here the authors establish human ventral midbrain organoids coupled with single cell sequencing to study developing and mature dopamine neurons and use silk scaffolding to generate bioengineered brain organoids

## Introduction

The ability to control cell-fate specification and drive differentiation of human pluripotent stem cells (hPSCs) into regionally specified neuronal subtypes has opened up new avenues of research into human-specific brain development, disease modeling, and cell-based therapies. hPSC differentiation is most routinely carried out in two-dimensional (2D) cultures, which poorly recapitulate the cellular composition and structural complexity of human brain. Although models of human neural development that better recreate the intricacy of different regions of the developing neural tube are being developed^1–3^, it remains challenging to obtain mature cells in these systems. Three-dimensional (3D) human-brain organoids have rapidly become a widely adopted system to study the development and function/dysfunction of neuronal cells, as it provides a more physiologically relevant cellular context and allows for long-term maintenance of functionally mature neurons^4–6^. Recent advancements in single-cell sequencing technologies have increased the scope for dissecting organoid cultures to define cell-type composition and provide an opportunity to study brain development, cell diversity, and gene regulation of otherwise inaccessible human cells. To date, most studies of this sort have been conducted in cerebral organoids. For example, single-cell transcriptomics were used to map cell types and developmental states within organoids^7^, to explore cortical development^8^, and to define human-specific gene-regulatory changes^9^. However, the full potential of organoid models is still restricted by issues of reproducibility and variability in terms of morphology, cellular makeup, and activity^10–13^.

In this study, we patterned hPSC-derived brain organoids into a ventral midbrain (VM) identity using a protocol that results in the formation of midbrain dopamine (DA) progenitors and of functionally mature DA neurons after transplantation in xenograft models of Parkinson’s disease (PD)^14^. DA neurons in these organoids exhibited mature electrophysiological properties, neuromelanin production, and the ability to release DA, confirming the long-term maintenance of functionally mature human DA neurons in 3D culture as previously reported in both hPSC-derived^15–18^ and neural progenitor-derived^19–21^ midbrain organoids. A time-course transcriptional analysis of human VM development and DA neuron differentiation at single-cell level revealed four populations of cells with high transcriptional similarity to VM floor-plate cells, followed by the stepwise emergence of neurons, vascular leptomeningeal cells (VLMCs), astrocytes, and oligodendrocytes. Importantly, we found that mature neurons and glia formed within the organoids displayed high similarity to the corresponding cell types in adult human midbrain^22^. The large number of cells within the DA neuron cluster (14,606 cells) enabled us to perform a detailed molecular dissection of mature human DA neurons not previously possible. We identified several molecularly distinct human DA neuron subgroups, similarly to recent observations in developing and adult mouse brain^23–25^.

These brain organoids showed a lower level of variability compared with self-organized 3D structures^4,7^, and a similar level of variability as other protocols based on extrinsic patterning factors^15,26^. To further reduce variability in the organoids, we used recombinant spider silk microfibers functionalized with full-length human laminin. These bioengineered silk-VM organoids reproduced key molecular aspects of DA neurogenesis but with more precise patterning and less organoid-to-organoid as well as intra-organoid variation. Furthermore, detailed functional and molecular analyses showed that the silk scaffolds reduced necrosis and supported neuronal maturation in all regions of the organoid. In sum, silk-fiber scaffolding is an experimentally straightforward method that does not require specialized equipment or technical expertise and can thus be widely implemented in organoid culturing.

## Results

### Key features of human VM development can be recapitulated in 3D culture

**Fig. 1:**
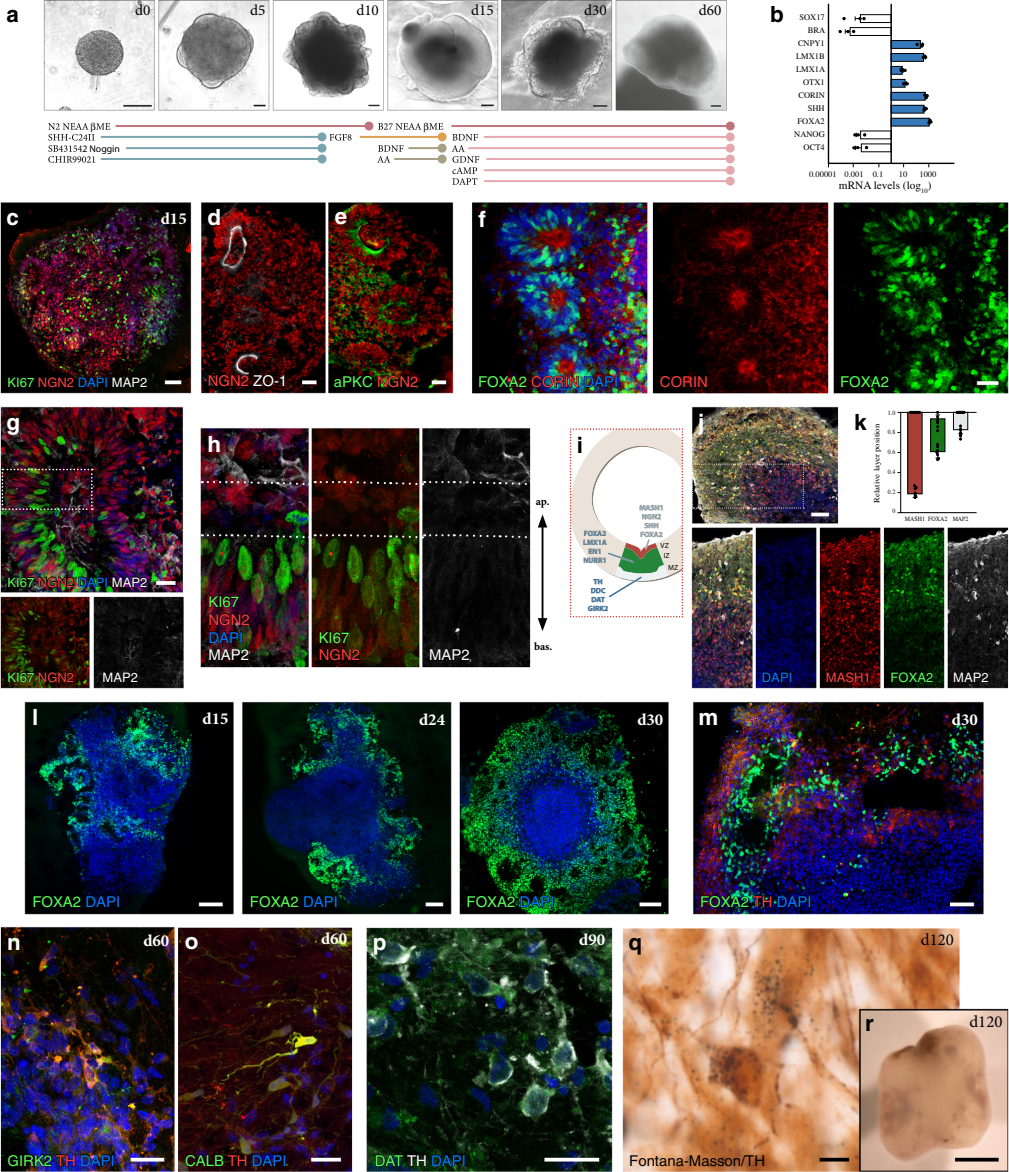
Dopamine neurogenesis in VM organoids. **a** Representative bright-field images of ventral midbrain (VM) organoid differentiation at different time points (upper) and schematic overview of the experimental design (lower). Scale bars, 200 µm. **b** qRT-PCR of selected markers at day 15 of VM organoid differentiation. The results are given as fold change over undifferentiated hPSCs. Data represent mean ± SEM of 3 independent organoids. **c**–**e** Immunocytochemistry of (**c**) NGN2/Ki67/MAP2, (**d**) NGN2/ZO-1, and (**e**) NGN2/aPKC at day 15 during VM organoid differentiation. Scale bars, 50 µm. **f** Immunohistochemistry of FOXA2/CORIN and **g** NGN2/Ki67/MAP2 in VM organoid at day 15. Scale bars, 100 µm (**f**) and 50 µm (**g**). **h** High-power magnification of the inset in **g**. **i** Schematic representation of developing DA neurons in vivo showing genes expressed at different stages of development (MZ, mantle zone; IZ, intermediate zone; VZ, ventricular zone). **j** Immunohistochemistry of MASH1, FOXA2, and MAP2 showing layer-specific organization and **k** relative quantification of fluorescence staining in VM organoid at day 24. Data represent mean ± SEM of 7 independent VM organoids. Scale bar, 100 µm. **l** Immunohistochemistry of FOXA2 across a time course (day 15–30). Scale bars, 50 µm. **m** Cryosection of VM organoid at month 1 showing TH/FOXA2 double staining. Scale bars, 100 µm. **n**, **o** Immunohistochemistry of TH stained with GIRK2/CALB at day 60 and **p** with DAT at day 90. Scale bars, 50 µm (**n**, **o**) and 20 µm (**p**). **q** Fontana Masson/TH double-stained cryosection from long-term cultured VM organoid (month 4). Scale bars, 50 µm. **r** Representative bright-field image of VM organoid at month 4. Scale bars, 1 mm. Nuclei were stained with DAPI in **g**, **h**, **j**, **l**–**p**. Source data are provided as a Source Data file.

We established VM organoids by adapting a commonly used protocol for the generation of forebrain organoids^27^ with the addition of dual-SMAD inhibition combined with exposure to SHH, GSK3i, and FGF8 in the same temporal sequence used in 2D culture to promote neurogenic conversion of VM FP progenitors toward a DA neuron identity (Fig. 1a)^28,29^. We confirmed that in 3D cultures, this patterning regime also resulted in efficient neuralization and ventral midbrain differentiation as evaluated by expression of *CNPY1*, *LMX1a*, *LMX1b*, *OTX1*, *CORIN*, *SHH*, and *FOXA2* (Fig. 1b). The expression of midbrain genes was accompanied by complete downregulation of pluripotency-associated genes *OCT4* and *NANOG* (Fig. 1b). Expression of early neural marker NGN2 (Fig. 1c–e) as well as tight-junction protein ZO1 (Fig. 1d) and atypical protein kinase aPKC (Fig. 1e) was detected by immunocytochemistry at early stage of organoid formation. Although the architectural arrangements morphologically resembled the intermediate rosette stage typical of anterior neuroectoderm^30^, they were PAX6-negative (Supplementary Fig. 1a–c) and expressed both CORIN and FOXA2 (Fig. 1f), in accordance with their VM identity. Successful 3D VM differentiation was reproduced using two additional cell lines, H9 and HS1001 (Supplementary Fig. 1d–k), demonstrating the robustness of the protocol.

Staining for the cell cycle marker Ki67 showed that cell proliferation was largely confined to the basal region, while postmitotic neurons detected by MAP2 were located in the apical regions (Fig. 1g, h). We further assessed the presence of different developmental zones as defined by expression of MASH1, FOXA2, and MAP2 (Fig. 1i–k) and found a layered-restricted organization (Fig. 1j). This was confirmed by quantification of cells in different anatomical locations of the organoids, showing that FOXA2- and MAP2-expressing cells were concentrated to the outer layer (Fig. 1k). By the second week, FOXA2-positive cells appeared in the organoids (Fig. 1l), and from day 30, TH-expressing neurons were detected (Fig. 1m). To directly compare our VM organoids with other hPSC-derived midbrain-patterned organoids generated using previously published protocols, we recreated midbrain-like organoids (MLO) according to Jo et al.^15^ and dorsomorphin A82-01 midbrain organoids (DA-MO) according to Kwak et al.^17^ as well as forebrain organoids (FBO) as reported in Lancaster et al.^27^. Quantifications revealed a similar number of cells expressing the key VM markers FOXA2, LMX1, and TH in all three midbrain patterned organoids (Supplementary Fig. 1l–p), while PAX6 was only expressed in FBO (Supplementary Fig. 1a, p).

After 60 days, expression of G-protein-regulated inwardly rectifying potassium channel 2 (GIRK2) (Fig. 1n and Supplementary Fig. 1q), calcium-binding protein 1 (CALB1) (Fig. 1o and Supplementary Fig. 1r), and DA transporter (DAT) (Fig. 1p) indicated that mature DA neuron subtypes had emerged within the organoids. Quantifications at day 60 confirmed comparable differentiation into mature DA neurons as from previously reported protocols (Supplementary Fig. 1q–w). Specification toward a mature and authentic A9-like DA population, which consists of pigmented neurons located in SNc in primate VM—and which is prevalently lost in PD—was corroborated by combined Fontana–Masson staining/TH immunohistochemistry revealing the presence of intracellular and extracellular neuromelanin, visible as dark granular pigmentation after long-term culture (Fig. 1q, r).

### scRNAseq reveals cellular composition and developmental trajectory in VM organoids

**Fig. 2:**
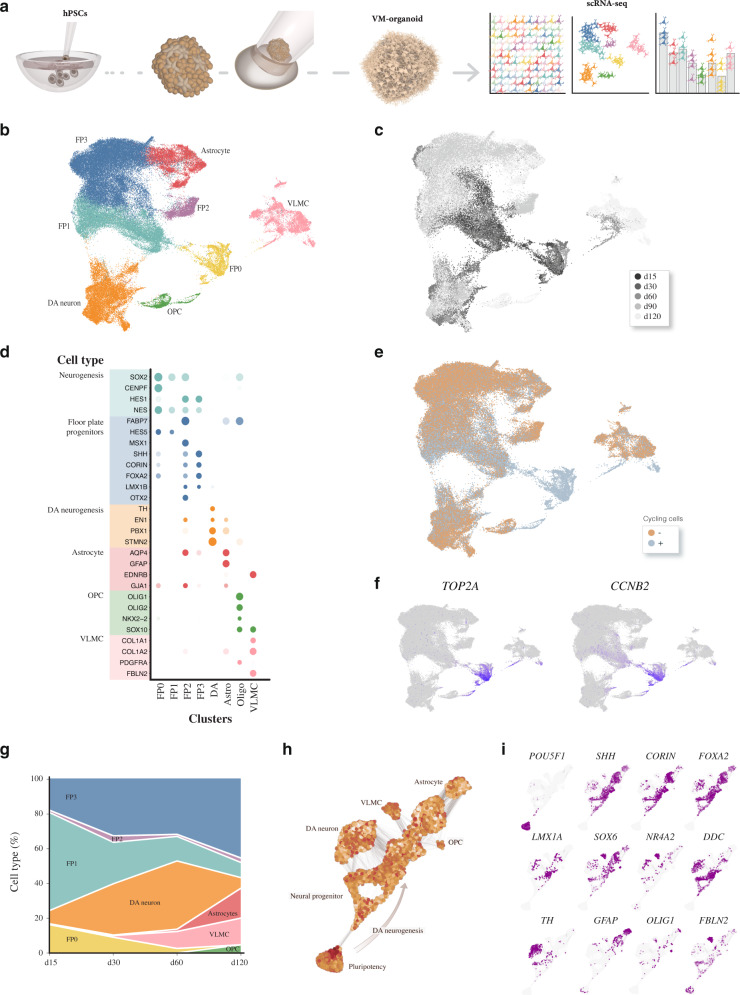
Single-cell transcriptomics identifying VM organoid cell types. **a** Schematic overview of the experimental design. hPSCs differentiated into regionalized VM organoids for up to 120 days, were analyzed at single-cell resolution. **b** 2D scatterplot of uniform manifold approximation and projection (UMAP) embeddings showing clustering of 91,034 analyzed cells from VM organoids at days 15, 30, 60, 90, and 120. Cell-type assignments are indicated. **c** UMAP plot of cells color-coded by organoid of origin. **d** Dot plot showing expression levels of indicated genes in each cluster. Indicated genes are established markers for neural progenitors, floor-plate progenitors, DA neurons, astrocytes, oligodendrocyte progenitors (OPCs), and vascular leptomeningeal cells. **e** UMAP plots showing cell cycle classification of analyzed cells (Seurat CellCycleScoring predictions). Cycling cells shown with gray dots. **f** Expression levels of indicated cell cycle genes visualized in the UMAP plots. **g** Proportion of each cell type along the temporal axis during VM organoid differentiation (day 15–120). **h** Developmental trajectory from pluripotency to terminally differentiated stages in VM organoid reconstructed using SPRING in VM organoid. Pseudocells are color-graded by total count. **i** SPRING plot colored (purple) by marker gene expression in emerging cellular clusters.

We next performed a 10X genomics droplet-based single-cell time-course transcriptomic analysis of human VM organoid development (Fig. 2a) by profiling a total of 91,034 single cells isolated from organoids at days 15, 30, 60, 90, and 120 of VM organoid differentiation with several replicates per time point (day 15, *n* = 2; day30, *n* = 4; day 60, *n* = 5; day 90, *n* = 2; day 120, *n* = 6). After integration using the Harmony approach^31^, uniform manifold approximation and projection (UMAP) and graph-based clustering visualized eight different clusters (Fig. 2b), all assigned a neuroectodermal identity (Fig. 2b–d and Supplementary Fig. 2c) with no remaining pluripotent cells (Supplementary Fig. 3a), mesodermal (*T*), or endodermal derivatives (*SOX17*) (Supplementary Fig. 3b). All identified clusters showed distinct separations with high average silhouette widths (Supplementary Fig. 2b).

The most highly differentially expressed genes were used together with canonical markers to manually annotate clusters with their respective cellular identities. The yellow cluster in the UMAP space was made up of cells expressing neural markers (*HES1*, *NES*, and SOX2) (Fig. 2d and Supplementary Fig. 3c) and cells with a highly proliferative signature (*TOP2A, CCNB2,* and *CENPF1*) (Fig. 2e, f), which we named FP-0. These cells display similar characteristics to cycling FP progenitors located in VM at early stages of embryonic development^32^^,^^33^. FP-0 population was the prominent cluster at the early time points (Fig. 2c and Supplementary Fig. 4a, b), and based on pseudo-time-inference reconstruction analysis (Supplementary Fig. 4c) gave rise to all the other identified cell clusters (see later section). UMAP also visualized another large FP-like progenitor population that was further subdivided into three different clusters, referred to as FP-1 (light green), FP-2 (purple), and FP-3 (blue) (Fig. 2b). The concomitant expression of *HES5* and *SOX2* with *VIM* and *FABP7* (also known as *BLBP1*) (Fig. 2d and Supplementary Fig. 3c, d) indicated that FP-1 shares many features of radial glial cells and is also enriched in cell cycle genes (Fig. 2e). FP-2 was instead enriched in cells expressing *SHH* and *CORIN*, and also contained early DA progenitor markers, including *FOXA2*, *LMX1A*, *MSX1*, and *OTX2* (Fig. 2d and Supplementary Fig. 3e). The FP-3 cluster mainly differed from the FP-2 cluster in the additional expression of neuronal precursor markers *STMN2* and *SYT1*, and DA progenitor markers *EN1* and *DDC* (Fig. 2d and Supplementary Fig. 2c and Supplementary Fig. 3f, g), as well as its exit from cell cycle (Fig. 2e, f). Furthermore, scRNAseq analysis revealed the absence of forebrain (*FOXG1*) and only very few scattered hindbrain (*HOXA2*) cells, indicating efficient VM patterning (Supplementary Fig. 3d). In agreement, the neuronal cluster (orange in Fig. 2b) defined by expression of *DCX*, *SYT1*, and *STMN2*, primarily expressed genes associated with DA-fate identity (*PBX1, NR4A2/*NURR1*, EN1, TH*, and *DDC*) (Fig. 2d and Supplementary Fig. 2c and Supplementary Fig. 3f, g), with only few cells showing GABAergic (*VGAT*) and glutamatergic (*VGLUT1*) features (Supplementary Fig. 3k). VM organoids also contained a newly discovered class of perivascular-like cells termed vascular leptomeningeal cells (VLMCs) expressing *PDGFRa*, *COL1A1*, *COL1A2*, and *LUM*, astrocytes (*GFAP, AQP4,* and *EDNRB*) and a small cluster of oligodendrocyte progenitors (*OLIG1/2, PDGFRa,* and *SOX10*) (Fig. 2b, d and Supplementary Fig. 3h–j and l–n). Consistent with efficient VM patterning, the single-cell dataset generated here correlated well with published bulk and single-cell sequencing of midbrain 3D cultures derived from either hPSCs or neural progenitors (Supplementary Fig. 5a)^15,20,21^ and, as expected, displayed a lower correlation to FBO (Supplementary Fig. 5a). We also performed scRNAseq of one-month-old MLOs generated using the protocol described by Jo et al. (29,112 cells analyzed from two replicates), which showed the presence of the same cell types at the same timepoints (Supplementary Fig. 5b).

Analysis of organoid composition over time revealed that the different cell types appeared in a temporal pattern: the yellow cluster was the first emerging progenitor population, while FP-2 and FP-3 appeared slightly later (Fig. 2g and Supplementary Fig. 4a, b). Unlike FP-0 and FP-1, FP-3 increased proportionally in frequency over time and FP-3 was the only population still present in high numbers at day 120 (Fig. 2g and Supplementary Fig. 4a, b). The DA cluster started to emerge from day 15 and was present in high numbers at all subsequent time points analyzed, although its relative proportion varied due to the increased presence of other cell types at later timepoints (Fig. 2g and Supplementary Fig. 4a, b). VLMCs, astrocytes, and oligodendrocyte progenitors appeared in a sequential manner (Fig. 2g and Supplementary Fig. 4a, b). When organizing cells—from pluripotent to mature differentiated states—according to transcriptional similarity along a temporal axis, force-directed *k*-nearest-neighbor graph-based pseudotime trajectory defined distinct branches segregating from the FP progenitor cells (Fig. 2h, i). At month 1, a first branch gave rise to DA progenitors, which by month 2 had started to mature into postmitotic DA neurons (Fig. 2h, i). A second branch trajected toward vascular stromal progenitors able to differentiate into VLMC progenitors after month 1, but into more mature cell types only from month 3 (Fig. 2h, i). These findings were corroborated by Slingshot analysis (Supplementary Fig. 4c), where lineages are identified by treating clusters of cells as nodes in a graph and drawing a minimum spanning tree between nodes^34^.

### Molecular diversity in human DA neuron cluster

**Fig. 3:**
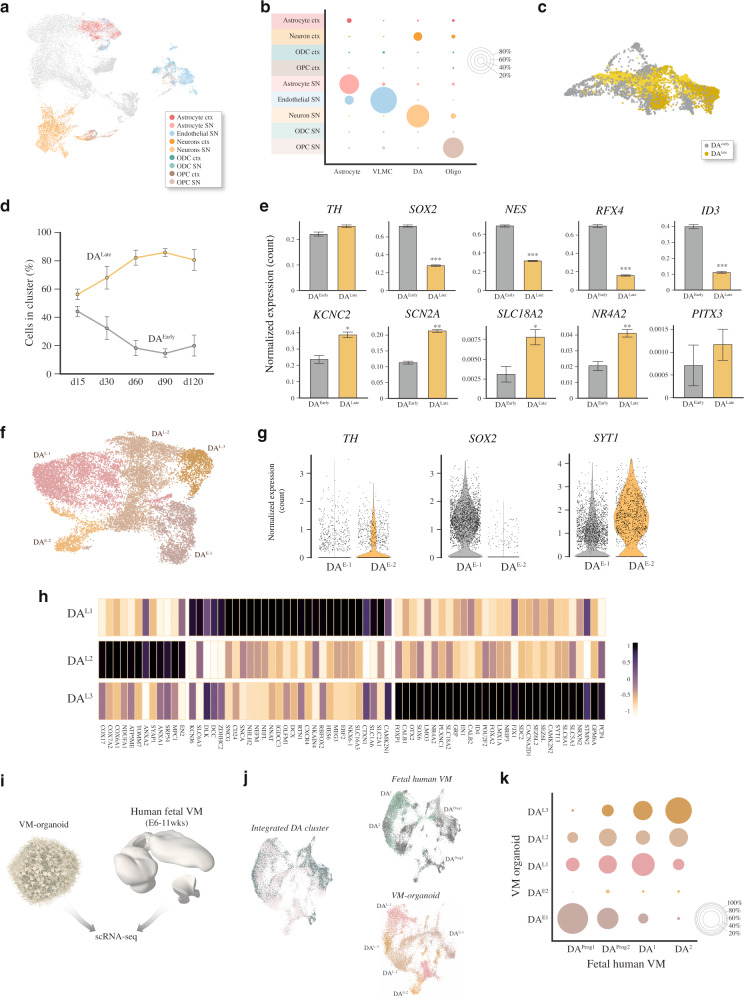
Single-cell transcriptomics mapping DA diversity in VM organoids. **a** UMAP cluster-integration analysis combining published scRNA-seq datasets of adult human midbrain^18^ and the hPSC-derived VM organoids with **b**, relative overlapping quantification. **c** SPRING network plot showing the distribution of single cells in 2 dopamine (DA) clusters (DA^Early^, gray, and DA^Late^, yellow) within the VM organoids. **d** Percent distribution of DA^Early^ and DA^Late^ clusters across a time course during VM organoid differentiation (d15 *n* = 2; d30 *n* = 5; d60 *n* = 5; d90 *n* = 2; d120 *n* = 6). Data represent mean ± SD per 10X run. **e** Bar plot of normalized expression for DA^Early^ and DA^Late^ clusters of immature and mature neuronal marker genes (d15 *n* = 2; d30 *n* = 5; d60 *n* = 5; d90 *n* = 2; d120 *n* = 6). Data represent mean ± SEM, two-tailed Wilcoxon Rank Sum test, KCNC2 *p* = 0.0045; SCN2A *p* = 0.0002; SLC18A2 *p* = 0.0269; NR4A2 *p* = 0.0013, ***p < 0.0001. **f** UMAP plot showing DA subclusters after reintegration and clustering (DA^E-1^, DA^E-2^, DA^L-1^, DA^L-2^, and DA^L-3^). **g** Violin plots showing differential expression levels of indicated genes in each DA^Early^ subclusters. **h** Heatmap showing differentially expressed genes and manually selected markers in 3 DA^Late^ neuron subclusters (DA^L-1^, DA^L-2^, and DA^L-3^). **i** Schematic overview of experimental design where scRNAseq data from dissected human fetal VM (6–11-week embryos) and 3D primary cultures thereof (1 month) Birtele et al., bioRxiv doi.org/10.1101/2020.10.01.322495. **j** Overlapping and individual UMAP plots showing DA subcluster-integration analysis from scRNA-seq dataset of human fetal VM and hPSC-derived VM organoid. **k** Relative overlapping quantification of DA organoid subtypes vs human fetal DA neuron dataset after prediction of DA neuronal subtypes using fetal data as reference (Seurat).

We next investigated whether the mature cell types generated in VM-patterned organoids were transcriptionally similar to those present in a recently published snRNA-seq dataset from adult human midbrain containing ∼6000 midbrain nuclei derived from five adult individuals^22^. This study identified distinct cell types in the adult midbrain: astrocytes, oligodendrocytes, oligodendrocyte progenitors (OPCs), microglia, endothelial cells, and neurons^22^. Merged clustering with our VM organoid dataset showed that the cell types present in organoids and human midbrain were transcriptionally similar, with the exception of microglia, which were not present in the organoids (Fig. 3a). Interestingly, all cell types in VM-patterned organoids displayed much higher transcriptional similarity to the corresponding cell types in human midbrain than to those in the cortex from the same dataset (~10,700 cortical cells from the middle frontal gyrus) (Fig. 3b).

Histological analysis (Fig. 1) indicated that DA neurons mature over time in the organoids, and that markers enriched in the two subtypes A9 and A10 neurons were present in long-term cultures (Fig. 1n–r). Several recent studies based on scRNAseq describe a greater-than-expected molecular DA neuron diversity, and at least five different molecular subtypes are reported in adult mouse VM^23–25,35^. However, similar datasets for mature human DA neurons do not yet exist. To determine if distinct molecular subtypes of human DA neurons appear in the organoids, we isolated the DA compartment (14,606 cells, all time points) and reran the integration (Harmony) and clustering. A SPRING plot visualized two major populations (Fig. 3c), one mostly present at early time points and one present at late stages of VM organoid differentiation, which we termed DA^Early^ and DA^Late^, respectively (Fig. 3c, d). Both DA^Early^ and DA^Late^ expressed TH (Fig. 3e). The expression of embryonic/early neural markers (*NES*, *SOX2*, and *RFX4*) in DA^Early^ confirmed their relatively immature neuronal state, whereas the expression of mature, postmitotic DA markers (NURR1*/NR4A2*, VMAT-2*/SLC18A2*) and voltage-gated potassium- and sodium-channel subfamily members (*KCNC2*, *SCN2A*) defined the more mature DA population (Fig. 3e).

The resulting network map (Fig. 3f) indicated transcriptional diversity within this cluster, prompting us to perform a higher-resolution analysis in order to detect the potential existence of human DA neuron molecular subtypes. The refined graph-based clustering segregated the early DA neurons into two distinct clusters (Fig. 3f): one with low TH (DA^E-1^) and one with high TH (DA^E-2^) (Fig. 3g). DA^E-1^ also showed increased expression of neural markers (*SOX2*), and reduced level of *SYT1* compared with DA^E-2^ (Fig. 3g). The late DA neurons segregated into three distinct molecular identities (named DA^L-1^, DA^L-2^, and DA^L-3^) (Fig. 3f). Within the DA^L-1^ subcluster we found concomitant expression of *DLK1*, *KCNJ6* (also known as *GIRK2*), *SLC6A3* (*DAT*), and *DCC*. Interestingly, *SNCG* and *SNCA* (encoding members of the synuclein family of proteins), glycoprotein CD24, and transcription factors *ZDHHC2* and *NHLH2*, which were all observed in this subcluster, were also found enriched in SNc from mouse bulk and scRNAseq datasets^36^ (Fig. 3h). Synapse-associated protein 1 (*SYAP1*) transcription factors and engrailed homeobox 2 (EN2) were significantly expressed in DA-^L-2^. This subcluster also expressed *ANXA1*, encoding for a calcium-dependent phospholipid-binding protein, recently found associated with SNc at different developmental stages^25,37,38^, and a large set of genes coding for components of respiratory electron-transport complexes (*COX17*, *NDUF,* and *ATP5ME*) as well as the brain mitochondrial receptor (*MPC1*) (Fig. 3h). The DA^L-3^ subcluster was molecularly defined by expression of *OTX2* and *CALB1*, markers of A10 DA neurons, while *DLK1* also appeared enriched in both DA^L-3^ and DA^L-2^ (Fig. 3h)^23,39^. Importantly, the DA^L-3^ cluster was also enriched in genes previously identified at single-cell level during mouse development up until adulthood, such as POU2F2 and ID4, as candidate regulators that may drive A10 subtype diversification (Fig. 3h). A set of genes associated with neuropsychiatric conditions, including Alzheimer’s disease (*CLU*, *P4HA1*), schizophrenia (*CNIH2*, *DKK3*), and autism-spectrum disorders (*SEZ6L*, *SDC2*), was found particularly upregulated in this cluster (Fig. 3h)^40^.

To assess to what extent the molecular identity of DA neurons in the organoids corresponds to that of authentic human DA neurons, we compared our single cell data with two scRNA-seq datasets of fetal VM DA populations, one previously reported by La Manno et al.^35^ (Supplementary Fig. 5c, d) and the other obtained from 6 to 11-week post-conception human embryos and cultures thereof (Birtele et al., bioRxiv doi.org/10.1101/2020.10.01.322495) (Fig. 3i). In this latter fetal dataset, containing more mature neurons, 18,848 human fetal DA cells formed four molecularly distinct DA populations, two of which consisted of DA progenitors (gray) and the remaining two more mature populations (green) (Fig. 3j and Supplementary Fig. 5e). To confidently define organoid DA populations, we integrated fetal and hPSC-derived data, and normalized and clustered the gene expression matrix, identifying commonalities visualized via UMAP (Fig. 3k). We found a high similarity between developing and mature DA cell populations in hPSC-derived subclusters and their fetal DA neuronal counterparts (Fig. 3k), demonstrating that DA neurons in VM organoids have a similar molecular identity to authentic midbrain DA neurons sourced from human fetal brain. A detailed comparison between the four clusters of fetal VM-derived DA neurons with the five clusters of DA neurons detected in VM organoids revealed that DA progenitors from fetal brain showed high transcriptional similarity to DA^E-1^ in the organoids, unlike DA^E-2^ (Fig. 3k). Moreover, DA^L1,2,3^ all showed high similarity to the mature fetal VM-sourced DA neurons (Fig. 3k).

### Molecular and functional heterogeneity in VM organoids

**Fig. 4:**
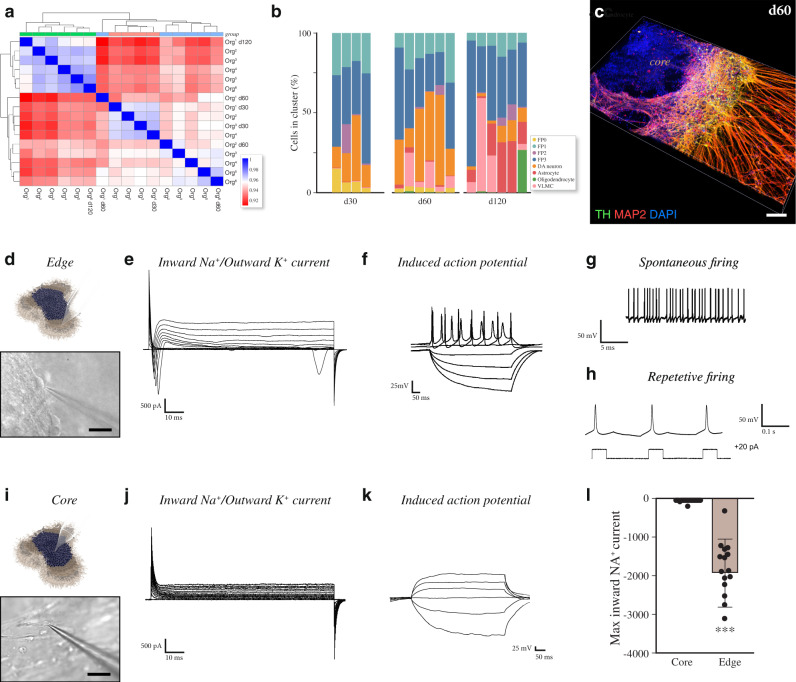
Molecular and functional heterogeneity in human VM organoids. **a** Clustering of sample to sample correlations (Pearson) of organoids and different timepoints using Euclidean distance on normalized and log‐transformed read counts. **b** Percentage of cells belonging to each cell cluster from individual organoids at days 30, 60, and 120. Intraclass correlation coefficient (correlation metric that considers group structure in the data) decreased from 0.717 at day 30 to 0.682 at day 60 and to 0.548 at day 120. **c** 3D reconstruction of an image stack from an 80 µm-thick optical section of TH and MAP2 immunohistochemistry at day 60. Scale bars, 100 µm. **d** Representative image of external functional recordings using whole-cell patch-clamp technique. Scale bars, 100 µm. **e** Representative trace from external patching showing inward sodium- and outward potassium-rectifying current traces of VM organoid at day 90 triggered by stepwise depolarization. **f** Patch-clamp recordings of external VM organoid cells depicting current-induced action potentials (APs) at day 90 (−85 pA to +165 pA with 20 pA steps). **g** External spontaneous firing at resting-membrane potential indicative of mature DA neuronal physiology in VM organoids at day 90. **h** Example trace of rebound depolarization after brief membrane depolarization (20pA) indicative of DA phenotype in externally located cells. **i** Representative image of internal functional recordings using whole-cell patch-clamp technique, Scale bars, 100 µm. **j** Representative inward sodium- and outward potassium-rectifying current traces of internally located cells at day 90 triggered by stepwise depolarization. **k** Patch-clamp recordings of internal VM organoid cells depicting the absence of current-induced APs at day 90 (−85 pA to +165 pA with 20 pA steps). **l** Inward sodium current quantifications in externally (*n* = 20) and internally (*n* = 16) localized cells within VM organoids at day 90. Data represent mean ± SD, unpaired two-tailed *t*-test, *p* = 0.0007. Source data are provided as a Source Data file.

scRNAseq followed by clustering of sample-to-sample correlations (Pearson) (Fig. 4a) and principal component analysis (PCA) (Supplementary Fig. 6a) revealed that organoids analyzed at the same developmental stage (days 30, 60, and 120) contained the same cell types (Fig. 4b), confirming the reproducibility of this protocol. However, relative frequency analysis quantifying changes in cell-type composition revealed high variability in the proportion of cell types within each cluster from organoid to organoid (Fig. 4b) even though the VM organoids were generated from a small number of hPSCs (2500 cells) following an optimized protocol reported to reduce organoid-to-organoid heterogeneity and increase long-term viability of 3D structures^7,41^. In addition to the variation between individual organoids, intra-organoid heterogeneity was observed in serial confocal TH-stained sections (Fig. 4c), showing that VM organoids exhibited a poorly developed core with sparse TH^+^ neurons, suggesting that nonsynchronous differentiation and maturation takes place. To test this, we performed whole-cell patch-clamp recordings to assess functional maturation of neurons within organoids using a recently reported method based on embedding in low-melting-point agarose^42^, allowing recordings in both the interior and exterior regions. We found that the cells at the surface of the organoids (Fig. 4d) exhibited more hyperpolarized resting-membrane potentials (Supplementary Fig. 6b, c) and rapidly inactivating inward sodium (Na^+^) and outward delayed-rectifier potassium (K^+^) currents (Fig. 4e) indicative of a mature neuronal state. In line with these findings, cells in the external part (*n* = 16) displayed the ability to fire induced action potentials (APs) upon current injections, indicating a neuronal function (Fig. 4f). These cells also showed spontaneous firing at resting-membrane potential (Fig. 4g) as well as a rebound depolarization (Fig. 4h) typical for DA neuron phenotype. In contrast, when recording from cells located in the inner region (Fig. 4i), no inward Na^+^ and outward K^+^ voltage-dependent currents or abortive APs were observed (*n* = 20 cells) (Fig. 4j–l). This distribution of functional cells located toward the edge of the organoids was confirmed using two additional protocols for PSC-derived midbrain organoids (Supplementary Fig. 6d–i).

### Generation and characterization of silk-bioengineered VM organoids

**Fig. 5:**
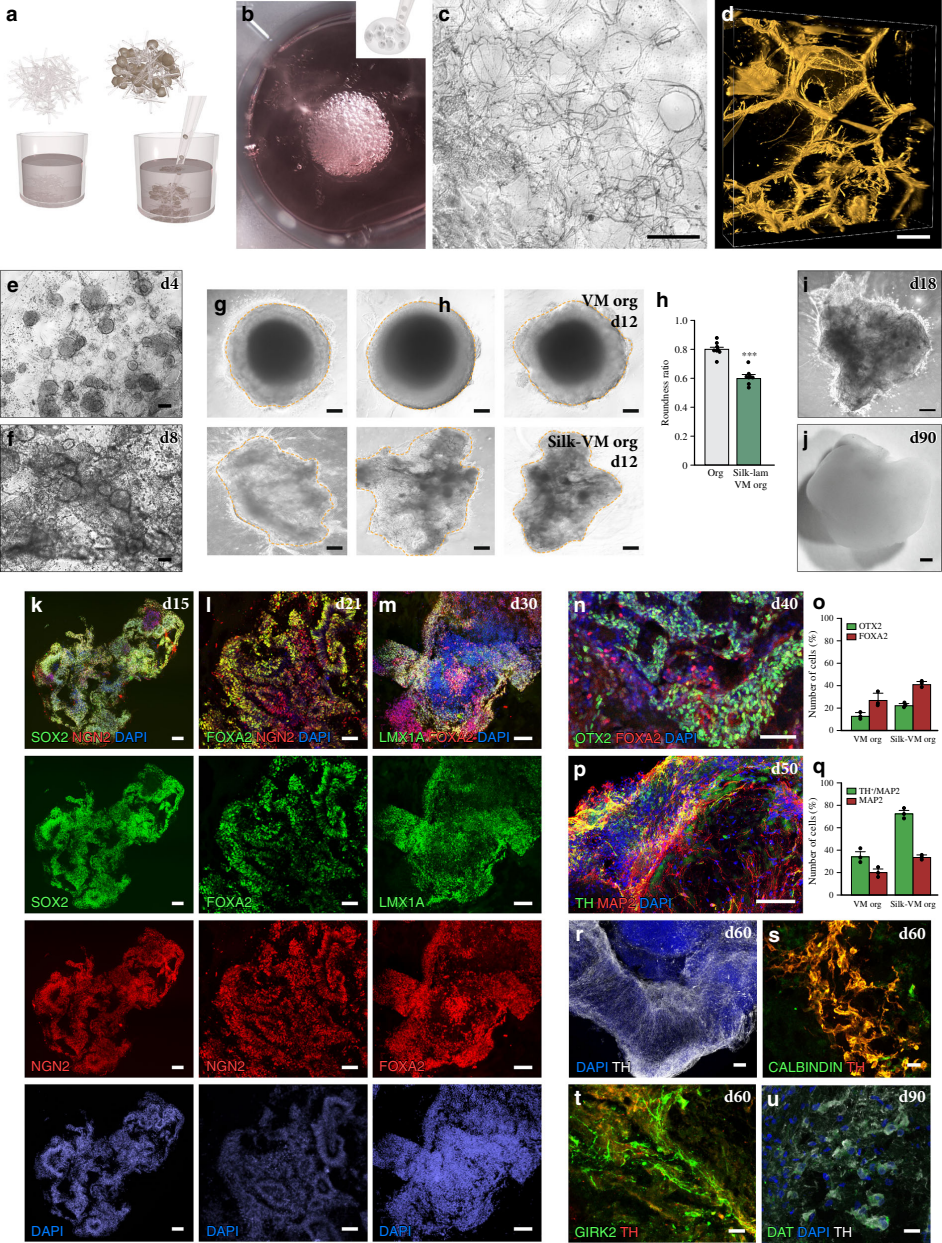
Generation and characterization of *silk*-VM organoids. **a** Schematic representation of *silk*-VM organoid generation. **b** Representative image of cells dispersed throughout silk foam. **c** Bright-field and **d** confocal images of 3D silk scaffold after reabsorption of foam. Scale bars, 50 µm. **e**, **f** Bright-field images showing adherence and growth of cells along the length of silk microfibers at days 4 and 8. Scale bars, 200 µm. **g** Representative bright-field images, and **h**, roundness measurement of VM organoids grown with and without scaffold at day 12. Scale bars, 100 µm. Data represent mean ± SEM of 8 biologically independent organoids, two-tailed Mann–Whitney test, ***p < 0.0001. **i** Representative bright-field images of a short-term and, **j**, long-term *silk*-VM organoid culture. Scale bar, 200 µm. **k** Immunohistochemistry of SOX2/NGN2 from organoid at day 15 and **l–n** FOXA2 across a time course from day 21 to 40, stained with NGN2 (**l**), LMX1A (**m**), and OTX2 (**n**). Scale bars, 100 µm (**l**, **n**) and 50 µm (**k**, **m**). **o** Quantifications of OTX2^+^ and FOXA2^+^ cells in VM and *silk*-VM organoids. Data represent mean ± SEM obtained from 3 independent organoids. **p** Immunohistochemistry of TH and MAP2 and **q**, quantifications of MAP2 and TH/MAP2 in VM and *silk*-VM organoids at day 50. Data represent mean ± SEM obtained from 3 independent organoids per condition. Scale bars, 100 µm. **r** Immunohistochemistry of TH and **s, t** with CALB1/GIRK2 at day 60. Scale bars, 20 µm. **u** Immunohistochemistry of TH stained with DAT at day 90. Scale bars, 20 µm. Nuclei were stained with DAPI in **j**–**l**, **m**, **o**, **q** and **t**. Source data are provided as a Source Data file.

In an attempt to create more homogeneous VM organoids and further reduce organoid-to-organoid variability, we evaluated a biomaterial made of recombinant spider-silk protein^43,44^ that can self-assemble into a biocompatible cell scaffold. Silk scaffolds in the form of a network of microfiber solution were obtained by placing a 20 μl droplet at the bottom center of a hydrophobic well and then introducing air bubbles by repeatedly pipetting air into the droplet (Fig. 5a and Supplementary Fig. 7a). Via self-assembly of the silk protein, a thin film was formed around each air bubble, producing a temporary foam (Fig. 5b and Supplementary Fig. 7a). hPSCs were then dispersed throughout the 3D silk scaffold to obtain the controlled cell distribution and adherence of cells within the network along the entire length of the microfibers (Fig. 5b and Supplementary Fig. 7b). Subsequently, during incubation in culture media, the foam collapses as the film around the air bubbles bursts, leaving a network of microfibers (Fig. 5c, d). Silk fibers were used either alone as an inert scaffold or functionalized with Lam-111, previously shown to promote DA patterning and support DA differentiation in 2D cultures^14,28^. With time, the cells gradually occupied the surface and inner space of the scaffold (Fig. 5e, f), and at day 10, the resulting 3D structures were mechanically detached from the bottom of the plate (Fig. 5g, h and Supplementary Fig. 7c, d). We named the resulting bioengineered organoids *silk-VM organoids* (Fig. 5i, j and Supplementary Fig. 7g). Unlike VM-patterned organoids grown without a scaffold, *silk*-VM organoids was less round and more variable in shape (Fig. 5g, h and Supplementary Fig. 7d), yet displayed a less distinct boundary between outer and inner regions (Fig. 5g, i and Supplementary Fig. 7e, f). Cell-viability assay indicated that the self-arrangement of cells along silk fibers to enhance organoid generation did not affect their viability (Supplementary Fig. 7c). Immunocytochemistry after one month confirmed a robust expression of the VM FP progenitor cell markers ZO-1, SOX2, FOXA2, and LMX1A (Fig. 5k–m and Supplementary Fig. 7h–l, n, o), indicating a similar developmental progression to that observed in organoids without scaffolding. The establishment of midbrain DA neuronal fate was confirmed by FOXA2 (Fig. 5n, o), TH, and MAP2 expression (Fig. 5p, q and Supplementary Fig. 7m, p–v). Quantifications at day 50 revealed a similar patterning and higher number of TH-expressing neurons in silk organoids (Fig. 5q and Supplementary Fig. 7t–v), followed by expression of GIRK2 and CALB1, markers of A9 and A10 DA neuron subtypes, and DAT at month 3 (Fig. 5r–u).

### Silk scaffolding reduces interorganoid variability in cell-type composition and DA neuron formation

**Fig. 6:**
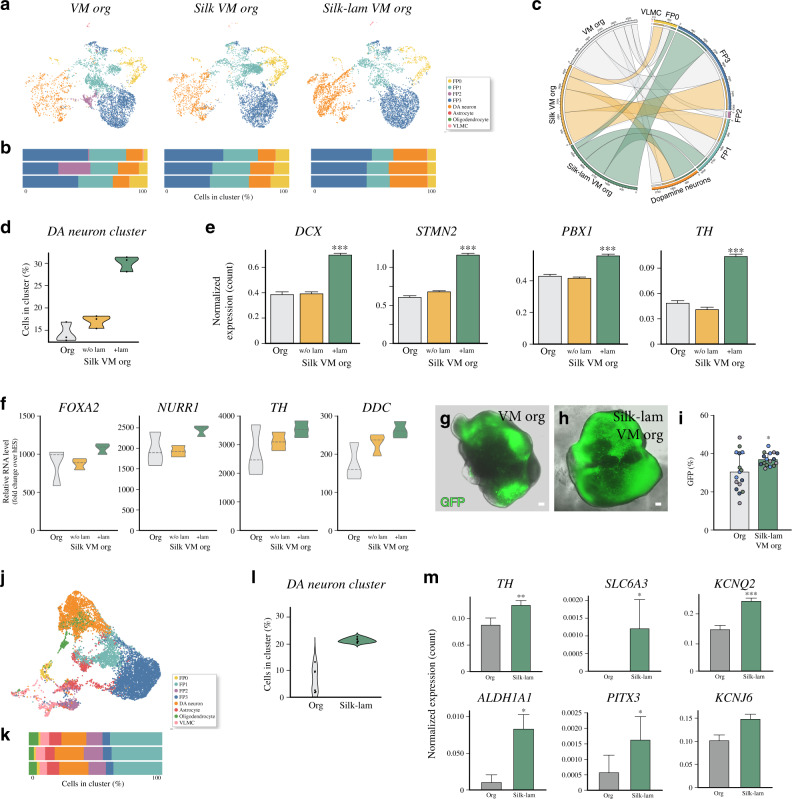
Single-cell transcriptomics identifying *silk*-VM organoid cell composition. **a** UMAP plots showing cell clusters from conventional VM, *silk*-VM, and *silk*-lam VM organoids and **b**, percentage of cells belonging to each cell cluster from individual organoids at month 1. **c** Chord diagram visualizing cell-type interrelationships between conventional, *silk*-VM and *silk*-lam VM organoids. **d** Violin plot showing the percentage of cells belonging to DA neuron clusters from conventional, *silk*-VM, and *silk*-lam VM organoids at month 1 from three individual organoids per condition. **e** Expression of selected markers belonging to DA neuron cluster in conventional, *silk*-VM, and *silk*-lam VM organoids at 1 month. Data represent mean ± SEM of 3 biologically independent organoids, two-tailed Wilcoxon Rank Sum test, ***p < 0.0001. **f** qRT-PCR analysis of early and late DA neuron markers in conventional and *silk*/*silk*-lam VM individual organoids at month 2. Data represent mean ± SEM of 3 independent organoids per condition. **g**, **h** Representative images of GFP expression in conventional and *silk*-lam VM organoids differentiated from the CRISPR/Cas9-mediated gene-edited *TH*-Cre hPSC line. Scale bars, 100 µm. **i** FACS-based quantification of GFP expression in conventional and *silk*-lam VM organoids differentiated from a CRISPR/Cas9-mediated gene-edited *TH*-Cre hPSC line in 4 biologically independent experiments shown as color-coded dots (green, light blue, blue and purple). Data represent mean ± SD, two-tailed unpaired *t* test *p* = 0.0162. **j** UMAP plot showing cell clusters from *silk*-lam VM organoids and **k**, percentage of cells belonging to each cell cluster from individual organoids at month 4. **l** Violin plot of percentage of cells belonging to DA neuron cluster from conventional and *silk*-lam VM organoids at month 4 from three individual organoids. **m** Expression of selected markers belonging to DA neuron cluster in conventional and *silk*-lam VM organoids at month 4. Data represent mean ± SEM of 3 biologically independent organoids, two-tailed Wilcoxon Rank Sum test, TH *p* = 0.0045, SLC6A3 *p* = 0.010462, KCNQ2 *p* = 0.0007, ALDH1A1 *p* = 0.046. Source data are provided as a Source Data file.

We used scRNAseq to compare 1-month-old VM organoids grown without a scaffold, here defined as “conventional” organoids (12,830 cells analyzed), silk-VM organoids (16,740 cells analyzed), and silk-VM organoids functionalized with Lam-111 (silk-lam VM organoids) (15,520 cells analyzed) from three independent biological replicates and separate 10X runs. UMAP embedding and graph-based clustering resulted in six major clusters (Fig. 6a). To annotate the clusters, we exploited the cell types identified in conventionally generated organoids and projected these labels onto the new data using Seurat’s v3 label transfer^45^. Frequency analysis quantifying the number of cells in each cluster showed a lower variability in both silk and silk-lam VM organoids than in organoids grown without a scaffold (Fig. 6b). UMAP plots (Fig. 6a) and chord diagram (Fig. 6c) visualizing cell-type interrelationships, revealed a larger DA cluster when the silk fibers were functionalized with Lam-111 (Fig. 6d, e and Supplementary Fig. 8a).

We next examined gene expression profiles of DA neurons and their progenitors in multiple independent batches of organoids generated with and without silk at month 2. Early and late DA markers were highly varied in inter- and intrabatches of conventionally generated VM brain organoids, as shown by RT-PCR, whereas self-arrangement into silk scaffolds alone or functionalized with Lam-111 significantly limited batch variability (Fig. 6f). To more precisely quantify the reproducibility of the silk methodology at protein level, we used a CRISPR/Cas9-mediated gene-edited transgenic hPSC line where *CRE* is knocked into the first exon of the *TH* gene^46^. When transduced with a flexed *GFP* lentiviral vector, this line serves as live reporter cell line where GFP is expressed specifically in DA neurons^46^. Also using this reporter system, the TH neurons appeared much more heterogeneously distributed in conventional vs silk organoids (Fig. 6g, h) Flow-based quantification in individual organoids established from this *TH*-Cre knock-in line revealed that *silk*-lam VM organoids displayed a higher percentage and more homogeneous formation of DA neurons (Fig. 6i and Supplementary Fig. 8b).

We further used scRNAseq to analyze three independent silk-lam VM organoid batches after four months. UMAP analysis of 18,375 cells visualized the same eight distinct major clusters (FP0–3, DA neurons, astrocytes, oligodendrocytes, and VLMCs) found in conventional organoids (Fig. 6j). However, frequency analysis quantifying the number of cells in each cluster revealed a lower variability in silk-lam VM organoids than in organoids grown without a scaffold also at this time point (Fig. 6k, l). To test this statistically, we utilized intraclass correlation (ICC), a correlation metric testing the proportions of each cell type in each 10X run where an ICC near 1.0 indicates high agreement. The sx batches produced using the standard protocol had an ICC of 0.51 (95% CI: 0.214–0.837) compared with 0.98 (0.96–0.99) for silk-lam VM organoids. Importantly, a high proportion of DA neurons was maintained long term in *silk*-lam organoids, indicating a precise and reproducible patterning (Fig. 6l). The FP2 population was also detected at this time point, suggesting that its absence at the early stage (Fig. 6a) reflects a slightly different developmental timing in silk organoids (Supplementary Fig. 8c). In addition, when analyzing the same number of cells and time points in both culture systems, DA neurons in silk-lam VM organoids showed a higher expression of TH and postmitotic DA neuron markers (Fig. 6m), indicating that a greater degree of maturation had been reached.

### Silk scaffolding reduces intra-organoid variability

**Fig. 7:**
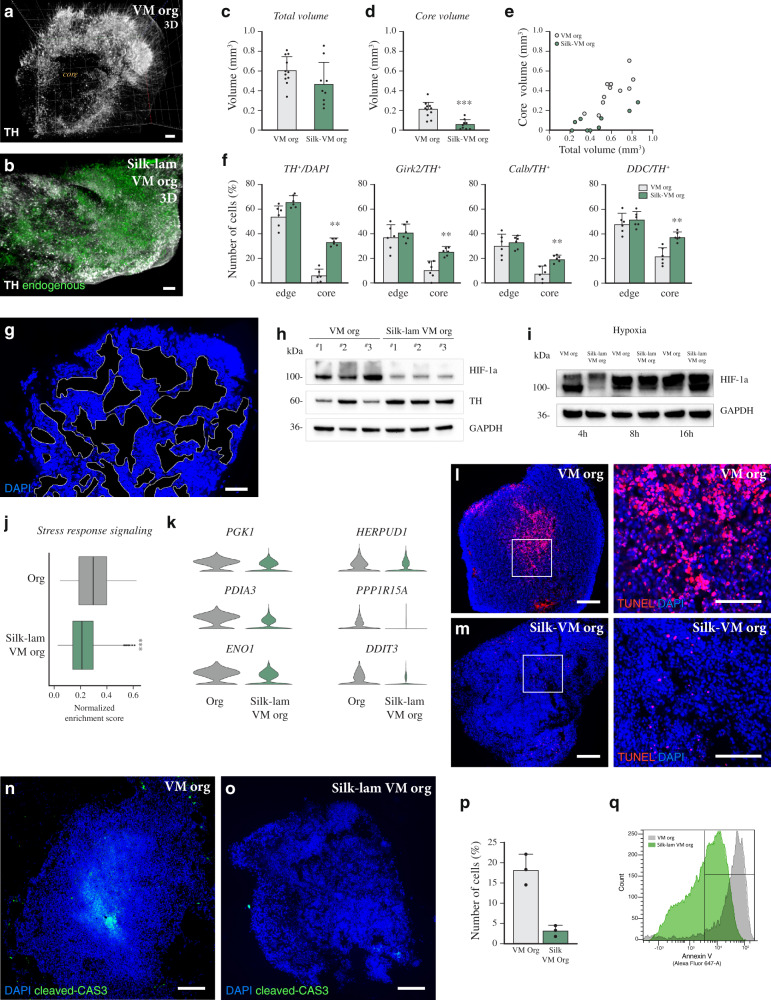
*Silk* fibers result in more homogeneous VM organoids. **a** iDISCO circuitry reconstruction obtained by mapping TH in conventional and **b** *silk*-lam VM organoids at day 60. Scale bar, 100 µm. **c** iDISCO-based total volume quantification and **d**, **e** core quantification of conventionally and *silk*-lam-generated VM organoids. Data represent mean ± SEM obtained from 11 and 9 independent conventionally and *silk*-lam-generated VM organoids respectively, two-tailed Mann–Whitney test, *p* = 0.0002. **f** Percentages of TH^+^, GIRK2^+^, CALB^+^, and DDC^+^ expressing cells located in the outer and inner layers in conventionally and *silk*-lam-generated VM organoids. Data represent mean ± SEM obtained from 6 biologically independent organoids per condition, two-tailed Mann–Whitney test, *p* = 0.002. **g** Immunohistochemistry showing microporous dimension. Scale bars 100 µm. **h** Representative Western blots of HIF-1α protein and TH expression in conventional and *silk*-lam VM organoids in normoxia conditions (21% O2). GAPDH was used as loading control. **i** Representative Western blots of HIF-1α protein in conventional and *silk*-lam VM organoids across a time course of 4 h, 8 h and 16 h under hypoxia conditions (<1% O2). GAPDH was used as loading control. **j** Gene Set Enrichment Analysis of Stress response signaling. Lower and upper hinges correspond to the first and third quartiles and the whisker extends from the hinge to the largest value no further than |1.5 * IQR | from the hinge (where IQR is the interquartile range, or distance between the first and third quartiles); two-tailed Wilcoxon Rank Sum test, ***p < 0.0001. **k** Representative markers of metabolic stress of DA neurons in VM organoids grown with and without scaffold at 4 months. **l**, **m** TUNEL staining of VM organoids grown with and without scaffold at 6 months. Scale bars, 100 µm. **n**, **o** Immunohistochemistry of cleaved caspase-3 and, **p** quantification of cleaved CAS3 over DAPI performed on conventional and *silk*-lam VM organoids at 6 months. Scale bars 100 µm. Data represent mean ± SEM of 6 biologically independent VM organoids per condition, two-tailed unpaired *t*-test, *p* = 0.0028. **q** FACS analysis for fluorescence intensity of Annexin-V staining in conventionally and silk-lam-generated VM organoids. Representative FACS plots of biological triplicates are shown. Nuclei were stained with DAPI in **g**, **j**. Source data are provided as a Source Data file.

Immunolabeling-enabled imaging of solvent-cleared organs (iDISCO) (Fig. 7a, b) reconstructed a more complex and highly intricate DA region throughout the entire silk-engineered organoids (Supplementary Video 1), showing more extensive DA circuitry and more efficient generation of VM regions than in conventional 3D cultures (Fig. 7c–e). Quantification of TH, GIRK2, CALB, and DDC in the core vs edge of organoids confirmed that the distribution of DA cells was much more homogeneous in *silk*-lam VM organoids than in our conventional VM organoids (Fig. 7f), as well as in previously described midbrain-patterned organoids (Supplementary Fig. 9a–d)^15,17^.

Studies on current organoid methods report that the increasing size of organoids can limit access to oxygen and nutrients in the inner layers, thereby affecting cell function and lifespan^12,47,48^. We found that while conventional and *silk* organoids had a similar volume (Fig. 7c), the volume of the immature core was smaller in *silk* organoids (Fig. 7d, e). Even when larger silk organoids were generated by proportionally increasing the number of cells and silk fibers, the core volume in each organoid remained small independent of the size of the individual organoid (Fig. 7c–e). By analyzing 2D sequential imaging of DAPI-stained sections, we observed the presence of porous microarchitectures in silk-engineered 3D structures (Fig. 7g) with an average cavity size of 3957 ± 817 µm^2^, likely to promote an increase in oxygen and nutrients in the inner regions and thus reducing cell death. To test this hypothesis, we performed whole-organoid Western blot analysis of hypoxia-inducible factor-1 alpha (HIF-1α), a key oxygen-labile protein. We found that silk scaffolding led to attenuation of hypoxic response pathway (Fig. 7h), which also persisted when *silk*-lam VM organoids were cultured for the first few hours under low oxygen tension (1%) in a gas-controlled chamber (Fig. 7i). Analysis based on scRNAseq showed that the global level of stress-response signaling in DA neurons is lower in silk than in conventionally generated VM organoids (Fig. 7j), as is the expression of individual genes associated with metabolic dysfunctions including glycolysis, oxidative stress and DNA damage (Fig. 7k). In agreement with this finding, decreased interior cell death was also observed in *silk*-lam VM organoids (Fig. 7l–q).

**Fig. 8:**
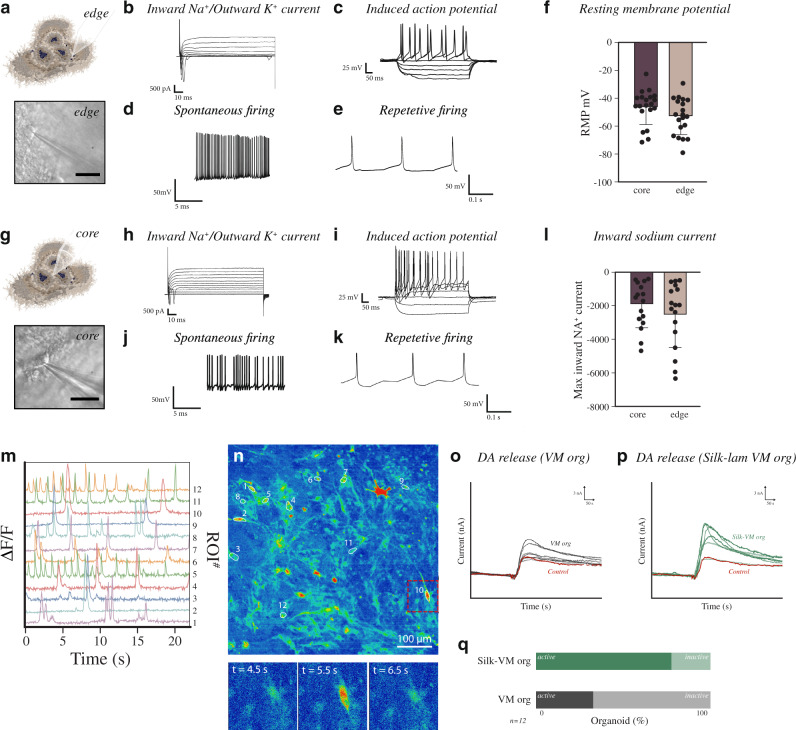
*Silk*-VM organoids are functionally homogeneous. **a** Representative images of functional recordings from the external part using whole-cell patch-clamp technique. Scale bars, 100 µm. **b** Representative inward sodium- and outward potassium-rectifying current trace of external VM organoid at day 90 triggered by stepwise depolarization. **c** Whole-cell patch-clamp recordings of external VM organoid cells depicting current-induced APs at day 90 (−85 pA to +165 pA with 20 pA steps). **d** Spontaneous firings at resting membrane potential indicative of mature DA neuronal physiology in *silk*-lam VM organoids in the external part at day 90. **e** Example trace of rebound depolarization after brief membrane depolarization (20 pA) indicative of DA phenotype in externally located cells. **f** Resting-membrane quantifications between externally (*n* = 20) and internally localized cells (*n* = 20) in VM organoids at day 90. Data represent mean ± SD. **g** Representative images of functional recordings from the internal region of organoid using whole-cell patch-clamp technique. Scale bars, 100 µm. **h** Representative internal inward sodium- and outward potassium-rectifying current trace of VM organoid at day 90 triggered by stepwise depolarization **i**, Whole-cell patch-clamp recordings in internal region of VM organoid cells depicting current-induced APs at day 90 (−85 pA to +165 pA with 20 pA steps). **j** Spontaneous firings at resting-membrane potential indicative of mature DA neuronal physiology in the internal region of *silk*-lam VM organoids at day 90. **k** Example trace of rebound depolarization after brief membrane depolarization (20 pA) indicative of DA phenotype in internally located cells. **l** Quantification of maximum inward sodium current recorded in internal (*n* = 16 cells) and external (*n* = 17 cells) regions. Data represent mean ± SD. **m** Differential fluorescence-intensity profile of intracellular Ca^+^ levels as a function of time in neurons expressing MAP2–GCamP5 at day 90. **n** Fluorescence image with marked regions of interest corresponding to recorded cells and three timeframes displaying the change in intracellular fluorescence intensity. Scale bar, 100 µm. **o**, **p** Representative analysis of real-time DA release chronoamperometric measurements in conventional and *silk*-lam VM organoids and **q**, relative quantification (*n* = 12).

Furthermore, the increased homogeneity, decreased cell death, and increased oxygen diffusion within the silk organoids resulted in mature and functional DA neurons in all regions of the organoid as assessed using whole-cell patch-clamp recordings from the outer and inner regions of the *silk*-VM organoids. This analysis revealed that in contrast to conventionally generated VM organoids, cells in the inner core of silk 3D culture also exhibited inward Na^+^ and outward K^+^ currents, confirming expression of voltage-gated sodium and potassium channels and a mature neuronal phenotype (*n* = 20 cells, inner; *n* = 19 cells, outer) (Fig. 8a–l and Supplementary Fig. 9e, f). Moreover, cells in both the core and outer layers of *silk*-lam VM organoids revealed mature electrophysiological properties of DA neurons with the presence of induced APs as well as spontaneous firing and rebound depolarization (Fig. 8b–e, h–k and Supplementary Fig. 9e, f). In addition, calcium imaging of MAP2–GcaMP5-labeled neurons indicated active neuronal signaling, confirming that mature and functional DA networks were present in silk-VM organoids (Fig. 8m, n). Finally, we performed real-time chronoamperometric measurements of DA exocytosis using a carbon-coated fiber (200 µm diameter) as a working electrode in a three-electrode electrochemical setup (Fig. 8o, p)^49^. Although DA release confirmed the high maturation and functionality of DA neurons in conventionally generated organoids, a lower proportion of the recordings showed a release of DA than in *silk*-lam VM organoids (Fig. 8q). Thus, although the quality of individual DA neurons generated in 3D organoids is comparable in conventional and silk organoids, the silk-based tissue engineering technology is more robust and results in better DA patterning with less variation within and between organoids.

## Discussion

We used VM organoids derived from pluripotent stem cells to perform a time course transcriptional analysis of human VM development and human DA neuron differentiation at single-cell level. This allowed us to (i) determine cellular composition, (ii) deduce the timing of cell-type appearance during organoid maturation, and (iii) trace developmental trajectories. We also more closely dissected the DA neuron cluster (14,606 cells), which led to the identification of three molecularly distinct subgroups of mature human DA neurons.

We used ventralizing and caudalizing factors known to direct a VM identity in 2D culture^28,29^ and to result in the formation of functional DA neurons after transplantation in preclinical rat models of PD^50,51^. A subset of these factors was previously used to generate similar midbrain-patterned organoids derived from hPSCs^15–17^ and other studies report VM organoids derived from neural progenitors^19–21^. Together, these reports show that it is possible to form and maintain DA neurons with mature functional properties for extended periods in 3D culture, and midbrain-patterned organoids have now started to be used for developmental studies, disease modeling, and high-throughput screening^18,20^.

In this study, we performed single cell transcriptional profiling of over 120,000 cells at different time points and produced a comprehensive map of cell type composition in VM organoids. This type of large-scale transcriptional mapping at single cell level has previously been performed in cerebral organoids, identifying a large diversity of neuronal cell types expressing both markers of multiple brain regions including forebrain, midbrain, hindbrain, and retina, as well as long-term excitatory/inhibitory neuronal identities^9,52^. Our analysis revealed that VM organoids have a more restricted repertoire of cell types, and that the composition is analogous to that observed in DAergic transplants from similarly patterned hPSCs, as previously described^53^. In addition, comparison of our single cell dataset with findings reported in adult human midbrain shows a high similarity between all mature cell types (neurons, astrocytes, and OPCS) within VM organoids and those present in human SNc, confirming the successful regionalization in vitro^22^ and supporting the use of midbrain organoids in developmental studies and in the therapeutic development.

The clear temporal appearance of different cell types, with FP-like cells being the dominant cell type at early time points followed by generation of DA neurons and subsequently development of astrocytes, OPCs, and VLMCs, enabled us to perform a detailed analysis of cell lineage using force-directed *k*-nearest-neighbor graph-based pseudotime trajectory to predict the future cellular states in each cluster. The readout of this analysis points to the fact that cells with an FP-like transcriptional profile serve as a common progenitor for all mature cell types arising in VM organoids. Further, the expression in both DA neuron and VLMC clusters of NR4A2 and SOX6, known to exert a critical role in the specification and maturation of DA phenotype, suggests that lineage segregation is a relatively late event during DA neurogenesis.

DA neurons in adult VM are traditionally divided into two main subtypes based on anatomical landmarks: A9 neurons located in SNc with projections into the dorsolateral striatum, and A10 neurons located in the ventral tegmental area (VTA) with more widespread projections including nucleus accumbens, septum, and prefrontal cortex^54,55^. Recent investigations at single cell level in mouse VM revealed a greater-than-expected molecular diversity in midbrain DA neurons^23,24,35^, but similar studies of human DA neurons are lacking. Previously, such a detailed analysis has not been possible using existing datasets from human midbrain^22,35^ due to the low number of mature DA neurons captured. To date, no scRNAseq data set from midbrain organoids obtained from pluripotent stem cells exists, and only one scRNA-seq analysis into neural progenitor-derived VM organoids is available in the literature^21^. This dataset^21^ describes a similar cell type composition to the one we identified in hPSC-derived VM organoids but did not analyze enough DA neurons or perform the late-stage profiling needed to define molecularly distinct DA neuron subtypes. The emergence of functionally mature DA neurons in high numbers within our VM patterned organoids derived using a protocol known to result in authentic and functional DA neurons capable of mediating functional recovery and circuitry reconstruction after transplantation in rat models of PD^28,50,51^, thus uniquely enabled a molecular analysis at single cell level in order to map human DA neuron diversity. By performing an unbiased and comprehensive characterization of the mature DA cluster we identified three molecularly distinct subtypes (DA^L-1^, DA^L-2^, and DA^L-3^). These separate DA clusters showed several points of similarity with previously published datasets from bulk and single cell mouse VM DA neuron populations^36^. We also identified a set of genes in VM organoids which may define similar DA neuron subtypes in both mouse and human. Our data suggest that DA^L-1^, which mainly expresses *SLC6A3*, *KCNJ6*, and *SNCG*, corresponds to two DA groups (T-Dat^high^ and AT-Dat^high^) reported to express high levels of DAT by Tiklova et al. and to another DA cluster (DA^1^) identified in adult mouse brain by Poulin and colleagues. *ANXA1*, which was found enriched together with *SLC6A3* and *MCP1* in our DA^L-2^ cluster, was used as a marker to distinguish SNc from VTA and to identify different SNc subpopulations during mouse VM development^25,37,38^. D^L-3^, characterized by *CALB*, *OTX2*, *LMO3*, and *SOX6* expression, seems to resemble other clusters, DA^2B^ and mDA2, previously described in two mouse studies by Poulin et al. and La Manno et al., respectively.

Our datasets provide insights into early fundamental regulators involved in molecular mechanisms that may play an important role in driving segregation of different mature DA neuron subtypes. A greater understanding of how subidentity is established could lead to the design of more targeted and effective DA neuron differentiation strategies, with implications for stem-cell-based therapies and disease modeling in PD. However, current organoid studies are often hampered by problems of reproducibility within and between organoids, as well as incomplete maturation resulting from interior hypoxia and the emergence of an immature or necrotic inner core^41^. Such issues were also observed in our study and in previous reports of VM organoids^15,19^. Interorganoid variability can be partially reduced by more precise patterning^20^, careful titration of the initial cell number^7^, starting with neural progenitor cells rather than pluripotent cells^19^, the adoption of scalable and automated culture systems^20^^,^^16^, or the use of scaffolds to guide self-organization^41^.

Bioengineering efforts to generate more homogeneous organoids with viable and mature cells in all regions are actively being pursued. Recent findings describe two very different strategies, one based on the creation of a 2D scaffold using individual inert microfilaments to guide the self-organization of hPSCs into organoids with more reproducible neuroectoderm features^41^ and the other using biomaterials such as hydrogels, which support bioengineered 3D neural cultures by mimicking the native brain extracellular matrix^56,57^. Exogenous vascularization in human organoids can also be achieved either by transplanting human organoids into a physiological environment in mouse brain^58^ and ectopically expressing genes to induce a vascular-like structure^59^, or by coculturing with endothelial cells^60^. These approaches at least partly address issues of reproducibility and maturation, but with limited experimental control. Moreover, the in vivo model of vascularized human brain organoids limits their use in large-scale biomedical applications such as drug screening, and the coculture or genetic induction of vessels may affect the directed differentiation of hPSCs necessary to obtain organoids of a specific brain region.

In this study, we used recombinant spider-silk microfibers that provide an easily accessible in vitro platform to generate bioengineered VM organoids, which we termed *silk*-VM organoids. These spider-silk microfibers present several advantages over existing methods using inert fibers. *First*, they are a recombinant chemically defined biomaterial that creates a strong, elastic, and biocompatible 3D scaffold able to guide the self-assembly of hPSCs into complex tissue-specific structures. *Second*, they can be easily functionalized with bioactive molecules that favor cell adhesion or control patterning. *Third*, they have the dual capacity to serve as both an anchored scaffold during early and a floating scaffold during late stages of organoid differentiation, allowing precise control of the ratio between cell density and number of fibers, thus decreasing variation between organoids. *Fourth*, silk scaffolds form porous microarchitectures, creating more favorable growth and differentiation conditions by allowing for the diffusion of oxygen and extrinsic patterning factors into the core, thereby reducing necrosis, reducing metabolic stress, and supporting neuronal maturation in all regions of the organoid.

*Silk*-VM organoids reproduce key molecular aspects of DA neurogenesis with a similar developmental progression pattern as conventionally generated organoids. The DAergic patterning in *silk*-VM organoids was further enhanced when functionalized with Lam-111. In addition, silk fibers sustain the homogeneous and functional generation of DA neurons throughout all compartments of the organoid in a highly efficient manner. Reduction of the necrotic core was previously achieved by mechanical cutting and generation of sliced human cortical organoid cultures to prevent hypoxia and cell death in the core^12^, or by engineering smaller organoids^16,20,59^. Unlike mechanically sliced organoids^12^, *silk*-VM organoids are preserved in size and shape, thereby more likely to maintain functional properties as well as the intricacy of neuronal networks. The remarkable properties of silk scaffolds combined with their straightforward use requiring no specialized equipment thus provide an easily accessible in vitro methodological platform able to generate organoids in a reproducible and functional manner.

## Methods

### hPSC culture and 2D differentiation

Undifferentiated RC17 (Roslin Cells, cat. no. hPSCreg RCe021-A), H9 (WiCell, cat. no. hPSCreg WAe009-A), HS999 and HS1001 (kindly provided by the Karolinska Institute), and *TH*-Cre hPSC cells were maintained on 0.5 μg/cm2 Lam-521 (BioLamina, #LN-521)-coated plates in iPS Brew medium (Miltenyi, #130-104-368) and were differentiated into 2D VM-patterned progenitors using our GMP-grade protocol^14^. All procedures were in accordance with the European Union directive and approved by the local ethical committee at Lund University.

### Human brain organoid culture

To start 3D VM organoid differentiation, RC17, H9, HS999, HS1001, and *TH*-Cre hPSC cells were detached from the culture dish with 0.5 mM Accutase (Thermo Fisher Scientific, #A1110501) to yield a single cell suspension. Differentiation was initiated by plating 2500 single cells in each well of a 96-well U-bottom plate (Corning, #CLS7007) in iPS Brew with 10 µM Y-27632 dihydrochloride (Miltenyi, #130-106-538), as previously described^27^. After three days in culture, embryoid bodies were transferred to differentiation medium consisting of 1:1 DMEM/F12:Neurobasal medium (Thermo Fisher Scientific, #21331020 and #A1371201), 1:100 N2 supplement (Thermo Fisher Scientific, #A1370701), 10 μM SB431542 (Miltenyi, #130-106-543), 150 ng/ml rhNoggin (Miltenyi, #130-103-456), 400 ng/ml SHH-C24II (Miltenyi, #130-095-727), and 1.5 μM CHIR99021 (Miltenyi, #130-106-539), and in the presence of 200 mM l-glutamine (Thermo Fisher Scientific, #25030081) and 10,000 U/mL penicillin-streptomycin (Thermo Fisher Scientific, #15140122). About 1% minimum essential medium nonessential amino acids (MEM-NEAA, Sigma-Aldrich, #M7145) and 0.1% 2-mercaptoethanol (Merck, #8057400005) were maintained for the entire differentiation period. On day 11, developing VM organoids were transferred to a 24-well plate containing 1:50 Neurobasal medium, B27 supplement without vitamin A (Thermo Fisher Scientific, #12587010), and 100 ng/mL FGF-8b (Miltenyi, #130-095-740). On day 14, 20 ng/mL BDNF (Miltenyi, #130-096-286) and 200 µM L-Ascorbic acid (Sigma-Aldrich, #A4403-100MG) were added. At this point, VM organoids were embedded in 30 µL droplets of Matrigel (BD Biosciences), as previously described^27^. From day 16 onward, 0.5 mM db-cAMP (Sigma-Aldrich, #D0627-1G) and 1 μM DAPT (R&D Systems, #2634) were added to the culture medium for terminal maturation for up to four months.

We define a batch of organoids as all organoids established at the same time and in the same dish.

Two additional midbrain-patterned organoids were also generated according to previously published protocols. Midbrain-like organoids (MLOs) were grown in tissue growth induction medium containing midbrain-patterning factors, as described in Jo et al.^15^. Midbrain organoids (MOs) were cultured with the addition of 2 μM dorsomorphin (Sigma), 2 μM A83-01 (Peprotech), 3 μM CHIR99021 (Miltenyi), and 1 μM IWP2 (Biogems), as reported by Kwak et al.^17^. Forebrain organoids were also generated using a whole organoid differentiation protocol^27^.

### Human embryonic tissue source

Human fetal tissue from legally terminated embryos was collected in accordance with existing guidelines with approval of the Swedish National Board of Health and Welfare and informed consent from women seeking elective abortions. To determine the gestational age of the embryos, the crown-to-rump length was measured and the embryo was staged according to week post conception.

### Hypoxic conditions

Both conventional and *silk*-VM organoids were cultured at 37 °C in 21% O_2_ and 5% CO_2_ in normoxia conditions. For hypoxia analysis, VM organoids were transferred to the hypoxia chamber (Binder CB160), which was filled with 1% O_2_ and 5% CO_2_ and mixed with N_2_, and collected after 4, 8, and 16 h.

### Cell-viability analysis

Cell viability was measured using the colorimetric CyQuant cell proliferation assay (Invitrogen), following the manufacturer’s instructions. Absorbance was analyzed at 480–520 nm, using 50,000 cells harvested from whole conventional and *silk-*VM organoids. Each organoid was analyzed in 6 replicates (i.e., 300 000 cells from each organoid) using Biochrom Asys Expert 96 Microplate Reader (Biochrom). Apoptosis was detected via flow cytometry after staining using the Alexa Fluor 647 Annexin V conjugate (BD Pharmingen, #A23204) and Click-iT plus Tunel assay (Invitrogen #C10617) according to the manufacturer’s instructions.

### Silk-scaffold preparation and hPSC integration

A 20 µL droplet of Biosilk protein solution (BioLamina, #BS-0101) either alone or functionalized with Lam-111 (BioLamina, #LN111), was placed in the center of hydrophobic culture wells in a 24-well plate (Sarstedt, #833922500). Recombinant human laminin-111 was purchased from Biolamina (Sweden) and added to the silk protein solution at a final concentration of 10 μg ml−1. The FN-silk/LN-111 mixture was incubated 10 min at RT before usage. About 10 µM Y-27632 dihydrochloride was added^44^. Air bubbles were introduced into the droplet by pipetting up and down (10–15 strokes), creating a dense foam. Multiple foams are generated in series with uniform shape and size (Supplementary Fig. 7b). Foam can also assume different dimension by increasing/reducing the volume of silk droplet giving rise to different organoid size in culture. RC17, H9, and HS1001 hPSCs were detached from the culture dish with 0.5 mM Accutase (Thermo Fisher Scientific, #A1110501) and prepared as a concentrated single cell suspension (20,000 cells/µL in iPS Brew medium). A total of 80,000 cells from the cell suspension were added to the silk foam and dispersed throughout by 6–8 additional pipette strokes^61^. The interaction between cells and silk microfibers was stabilized at 37 °C in an incubator for 20 min. Prewarmed iPS Brew medium containing 10 µM Y-27632 dihydrochloride was added to the foam-integrated cells.

### Silk-bioengineered VM organoid generation and morphological characterization

After three days, differentiation medium consisting of 1:1 DMEM/F12:Neurobasal medium, 1:100 N2 supplement, 10 μM SB431542, 150 ng/mL rhNoggin, 400 ng/mL SHH-C24II, and 1.5 μM CHIR99021 was added from day 0 to 10, following the same protocol used for generating conventional VM organoids. At day 10, the resulting 3D structures were mechanically detached from the bottom of the plate with a spatula and transferred to a 6-well plate (Corning, #3471) and grown in suspension (free floating). *Silk*-VM organoids were embedded in 30 µL droplets of Matrigel and cultured following the VM organoid-differentiation protocol described in the subsection “hPSC VM organoid differentiation”. Images were collected on phase-contrast inverted microscope (Olympus, #CKX31SF). Morphological classification (spherical/nonspherical) was performed in triplicate. Roundness measurements were based on bright-field images and calculated as the ratio between diameters of the largest inscribed and the smallest circumscribed circle of the organoid silhouettes (dotted line). Images were analyzed in ImageJ (NIH).

### Library preparation, sequencing, and raw data processing

For 10x Genomics single-cell RNA sequencing, single-cell suspensions were loaded onto 10x Genomics Single Cell 3′ Chips along with the mastermix as per the manufacturer’s protocol (https://support.10xgenomics.com/single-cell-gene-expression/index/doc/technical-note-chromium-single-cell-3-v3-reagent-workflow-and-software-updates) for the Chromium Single Cell 3′ Library to generate single cell gel beads in emulsion (GEMs, version 3 chemistry). The resulting libraries were sequenced on either a NextSeq500 or a NovaSeq 6000 with the following specifications Read1 28 cycles, Read2 98 cycles, and Index1 8 cycles using a 200-cycle kit. Raw base calls were demultiplexed and converted fastq files using cellranger mkfastq program (bcl2fastq 2.19/cellranger 3.0). Sequencing data were first preprocessed through the Cell Ranger pipeline (10x Genomics, Cellranger count v2) with default parameters (expect-cells set to the number of cells added to 10x system), aligned to GrCH38 (v 3.1.0), and the resulting matrix files were used for subsequent bioinformatic analysis.

### Bioinformatics analysis of sequencing data

Seurat (v 3.1 and R version 3.6.1) was applied to the scRNA data for downstream analysis of matrix files. Cells with at least 600 but no more than 12000 genes detected were kept for analysis. In addition, cells with more than 20% mitochondrial reads were excluded. After log-transformation, 4000 highly variable genes were identified using vst and z-transformed expression values followed by dimensionality reduction (PCA). To integrate data from different 10x runs, Harmony was applied using the R-package “Harmony” using individual 10X runs as grouping variable. Harmony converged after 9 iterations and corrected coordinates were used for downstream analysis. To identify clusters, Louvain clustering (resolution 0.4, Seurat) was applied to harmony embeddings. Differential expression analysis between clusters was carried out using the Wilcoxon rank sum test (Seurat) with genes with a FDR-adjusted *p*-value < 0.05 considered significant. Gene ontology overrepresentation analysis was performed using the enrichGO function in the clusterProfiler package (3.13) using MSigDB as the database. Gene Set Enrichment Analysis was performed using the R package escape https://www.nature.com/articles/s42003-020-01625-6. For silhouette and tree analysis the cluster package (version 2.1) was used. Lineage inference and pseudotime reconstruction was performed using Slingshot (version 1.6.1). We first used the expression data from 4000top variable genes to generate the minimum spanning tree of cells in a reduced-dimensionality space (Harmony-corrected UMAP embeddings). Global lineage structure was identified with a cluster-based minimum spanning tree and fitting simultaneous principal curves describing each lineage using “slingshot” function. Pseudotime analysis was also performed using a force-directed layout of k-nearest-neighbor graphs (SPRING, http://pubmed.ncbi.nlm.nih.gov/29228172/) on normalized expression counts using default settings.

### qRT-PCR

Total RNAs were isolated using the RNeasy Micro Kit (QIAGEN#74004) and reverse transcribed using random hexamer primers and Maxima Reverse Transcriptase (Thermo Fisher #K1642, Invitrogen). cDNA was prepared together with SYBR Green Master mix (Roche#04887352001) using the Bravo instrument (Agilent) and analyzed by quantitative PCR on a LightCycler 480 device using a 2-step protocol with a 60 °C annealing/elongation step. All quantitative RT-PCR (qRT-PCR) samples were run in technical triplicates and the results are given as fold change over undifferentiated hPSCs using each of the two housekeeping genes for normalization (ACTB and GAPDH). Details and list of primers are reported in Supplementary Table 1.

### Organoid cryosectioning and immunofluorescence

Both conventional and *silk*-VM organoids were fixed in 4% paraformaldehyde for 5 h at 4 °C followed by washing in PBS three times for 10 min. Both conventional and *silk*-VM organoids were left to sink in 30% sucrose overnight. Sucrose solution was replaced with 1:1 OCT:30% sucrose mixture for 6 h and then transferred to a cryomold and filled with OCT. The embedded tissue was frozen on dry ice and either cryosectioned at 20 μM or stored at −80 °C. For immunohistochemistry, sections were washed in PBS1X for 10 min and then blocked and permeabilized in 0.3% Triton X-100 and 5% normal donkey serum in PBS1X. After incubation with primary antibodies, the sections were incubated for 1 h with the appropriate secondary antibodies (Alexa Fluor 488, 594, and 647 used at 1:400, Molecular Probes) and then mounted on gelatin-coated slides and coverslipped with PVA-DABCO containing DAPI (1:1000). A list of primary antibodies is reported in Supplementary Table 2.

Quantification of fluorescence for developmental layers identified was performed using Image J software (NIH, v1.49). Measurements were performed by taking a radial line-intensity profile (80 µm width) for each channel from the center of the organoid to the edge of the organoid, subtracting the background and recording the position where the positive signal begins and ends. Recordings were normalized to the length of the radial line and mean value with standard deviation plotted for each of three fluorescence channels.

Whole silk- and conventionally generated VM organoids were sequentially sectioned to obtain slices from edge to core. For quantifications, each section was quartered into 4 equal areas and then scanned using a confocal microscope under 20X magnification. The number of TH^+^, Calb^+^, Girk2^+^, and DDC^+^ cells was manually counted in each area using Image J software (NIH, v1.49), and summed to give the total number of positive cells per slice. Counting was performed on every 3 sections and the final counts were corrected for the total number of sections per organoid.

### Immunohistochemistry and neuromelanin staining

For diaminobenzidine (DAB) staining, the sections were incubated with secondary biotinylated horse antibodies diluted (1:200, Vector Laboratories) for 1 h at room temperature (RT), washed three times, and then incubated with avidin-biotin complex (ABC) for 1 h at RT for amplification. Peroxidase-driven precipitation of DAB was used to detect the primary antibody. In this step, the sections were incubated in 0.05% DAB for 1–2 min before addition of 0.01% H_2_O_2_ for a further 1–2 min. After development of DAB staining, the sections were placed in an ammoniacal silver solution to detect neuromelanin using a Masson Fontana Stain Kit (Atom Scientific, #RRSK12-100), according to the manufacturer’s instructions. The sections were then mounted on gelatin-coated slides and dehydrated in an ascending series of alcohol concentrations cleared in xylene, and coverslipped with DPX mounting.

### iDISCO

Both conventional and *silk*-VM organoids were fixed in 2% paraformaldehyde overnight at 4 °C and permeabilized in 0.2% Triton X-100 20% DMSO and then in 0.1% Triton X-100, 0.1% Tween20, 0.1% C_24_H_39_NaO_4_, 0.1% NP40, and 20% DMSO overnight at 37 °C. After incubation with primary antibodies for 2 days at 37 °C, the organoids were incubated for 2 days with the appropriate secondary antibody, embedded in 1% agarose, and dehydrated in an ascending series of methanol concentrations and dichloromethane as previously described^62^. Samples were imaged in a chamber filled with DBE. The cleared brain organoids were imaged on an Ultra Microscope II (LaVision Biotec) equipped with an sCMOS camera (Andor Neo, model 5.5-CL3) and 12x objective lenses (LaVision LVMI-Fluor 4x/0.3 or 12x/0.53 MI Plan). We used two laser configurations with the following emission filters: 525/50 for endogenous background and AlexaFluor 488. Whole-organoid volumes were quantified based on endogenous background, and DA neuron number based on TH+ cell. Several stacks (mosaic acquisition) were taken with 10% overlap to cover the entire brain-organoid volume, including edge and core. Stacks were acquired with ImspectorPro64 (LaVision Biotec) using 3 μm z-steps to acquire the volume in 3D. These image stacks were stitched to visualize the brain organoid in 3D with Arivis Vision 4D 3.5.0 (Arivis AG). The high density of cells in the core volume, characterized by the absence of TH+ staining, was quantified based on 525/50 endogenous signal. Rendered movies were compiled in Final Cut Pro 10.4.3 (Apple Inc.).

### Microscopy

Images were captured using an Epson Perfection V850 PRO flatbed scanner, a Leica DMI6000B widefield microscope, or a Leica TCS SP8 confocal laser-scanning microscope, or a Nikon inverted Ti2 microscope equipped with a CSU-W1 spinning-disk system. Image acquisition software was Leica LAS X and images were processed using Volocity 6.5.1 software (Quorum Technologies) and Adobe Photoshop. Any adjustments were applied equally across the entire image, and without the loss of any information.

### Flow cytometry

*TH*-Cre hPSC-derived single-cell suspensions, differentiated either from conventional or *silk*-VM organoids, were obtained using the Papain Kit (Worthington, #LK003150). GFP^+^ cells were analyzed with BD FACSAria III (BD Biosciences) and all data plots were generated using FlowJo software.

### Western blotting

VM-organoid lysates were prepared with ice-cold immunoprecipitation assay (RIPA) lysis buffer. Whole-cell lysates were then separated on gels (Invitrogen, NuPAGE 4–12%) and transferred to a PVDF membrane (Millipore, Immobilon-P Membrane, 0.45 μM). Detection was performed with ECL reagents (Amersham Biosciences). Details and list of antibodies are reported in Supplementary Table 2.

### Organoid-slice preparation and electrophysiology

About 3% low-melting-point agarose (Promega, #V2111) was dissolved and melted in Neurobasal medium at 37 °C and placed in a cubic PDMS mold (1 cm^2^). At day 90, VM organoids were immersed and embedded in an agarose mold and left to solidify for 5–10 mins at RT. Sections of 400 μm thickness were prepared using a vibratome tissue slicer (Leica VT1000 S) at 0.1 mm/s speed and 1 mm vibration amplitude. Agarose blocks containing the VM-organoids were submerged in oxygenated artificial cerebrospinal fluid (ACSF) at 4 °C during the cutting procedure. VM organoid slices were equilibrated for 30 min in oxygenated ACSF at 37.5 °C prior to whole-cell patch clamp recordings ^42^.

Whole-cell patch-clamp electrophysiological recordings were performed at day 90 of VM organoid differentiation. Both conventional and *silk*-VM organoids were transferred to a recording chamber containing Krebs solution gassed with 95% O_2_ and 5% CO_2_ at RT and exchanged every 20 min during recordings. The standard solution was composed of (in mM) 119 NaCl, 2.5 KCl, 1.3 MgSO_4_, 2.5 CaCl_2_, 25 glucose, and 26 NaHCO_3_. For recordings, a Multiclamp 700B Microelectrode Amplifier (Molecular Devices) was used together with borosilicate glass pipettes (3–7 MOhm) filled with the following intracellular solution (in mM): 122.5 potassium gluconate, 12.5 KCl, 0.2 EGTA, 10 HEPES, 2 MgATP, 0.3 Na_3_GTP, and 8 NaCl adjusted to pH 7.3 with KOH, as previously described^29^. Data acquisition was performed with pCLAMP 10.2 software (Molecular Devices); current was filtered at 0.1 kHz and digitized at 2 kHz. Cells with neuronal morphology and round cell body were selected for recordings. Resting-membrane potentials were monitored immediately after breaking-in in current-clamp mode. Thereafter, cells were kept at a membrane potential of −60 mV to −80 mV, and 500 ms currents were injected from −85 pA to +165 pA with 20 pA increments to induce action potentials. For inward sodium and delayed rectifying potassium current measurements, cells were clamped at −70 mV and voltage-depolarizing steps were delivered for 100 ms at 10 mV increments. Spontaneous action potentials were recorded in current-clamp mode at resting membrane potentials.

### Calcium imaging of MAP2–GCamP5-labeled neurons

Calcium imaging was performed at day 120 of VM cultures containing the MAP2‐GCamP5 reporter. Imaging was performed on an inverted Ti2 microscope (Nikon) equipped with a CSU‐W1 spinning-disc system (Yokogawa), a sCMOS camera (Teledyne Photometrics), and a 20 × objective. An environment control chamber was used to maintain the temperature at 37 °C and CO_2_ level at 5% during imaging. Exposure time was set to 50 ms Spontaneous activity was recorded from 3 different silk-lam VM organoids. Images were analyzed in ImageJ (NIH).

### Chronoamperometry

Three-electrode setup with a pyrolyzed carbon fiber as the working electrode was used to detect dopamine. Electrode fabrication, electrochemical characterization, and setup assembly were performed as previously described^49^. In short, to perform the measurements, an organoid was placed on the working electrode immersed in baseline buffer, a constant potential was applied, and the resulting current monitored. Dopamine release was chemically triggered through addition of stimulation buffer that elevated K+ concentration to 150 mM. Measured current was normalized to the baseline value and plotted with respect to time.

### Statistics and reproducibility

Statistical analysis of qRT-PCR data and immunofluorescence-based quantifications was performed using two-tailed Student’s *t*-test and *p*-values <0.05 were considered significant. For comparisons of electrophysiological properties, two-tailed Student’s t-test and one-way analysis of variance (ANOVA) was used. Data were statistically analyzed with the GraphPad Prism 9 software and presented as mean ± SEM, except where stated otherwise. Statistical analysis of sequencing data was conducted using two-tailed Wilcoxon rank-sum test (Seurat v3.1) in R v3.6.1. Please refer to the bioinformatics analysis section above for more details. Immunohistochemical staining images are representative of 6–12 sections from at least 4 biologically independent organoids. Western blots are representative of 3 biological replicates.

### Reporting summary

Further information on research design is available in the Nature Research Reporting Summary linked to this article.

## Supplementary information

Supplementary Information

Description of Additional Supplementary Files

Supplementary Video 1

Reporting Summary

## Data Availability

The scRNA-seq data generated in this study have been deposited in the Gene Expression Omnibus under accession code “GSE168323”. A reporting summary for this article is available as Supplementary Information file. All other relevant data supporting the key findings of this study are available within the article and its Supplementary Information files or from the corresponding author upon reasonable request. Source data are provided with this paper.

## Source data

source data electrophysiology files Fig4,8
source data electrophysiology files S6
source data files

## Code availability

The code used for this paper is available on GitHub https://github.com/ParmarLab/scRNA-seq_silk_organoids with 10.5281/zenodo.5603294.
