# Author Correction: Single-cell transcriptomics captures features of human midbrain development and dopamine neuron diversity in brain organoids

**DOI:** 10.1038/s41467-022-31024-w

**Published:** 2022-06-08

**Authors:** Alessandro Fiorenzano, Edoardo Sozzi, Marcella Birtele, Janko Kajtez, Jessica Giacomoni, Fredrik Nilsson, Andreas Bruzelius, Yogita Sharma, Yu Zhang, Bengt Mattsson, Jenny Emnéus, Daniella Rylander Ottosson, Petter Storm, Malin Parmar

**Affiliations:** 1 Developmental and Regenerative Neurobiology, Wallenberg Neuroscience Center, and Lund Stem Cell Center, Department of Experimental Medical Science, Lund University, Lund, Sweden; 2 Regenerative Neurophysiology, Wallenberg Neuroscience Center, Lund Stem Cell Center, Department of Experimental Medical Science, Lund University, Lund, Sweden; 3 Department of Biotechnology and Biomedicine (DTU Bioengineering), Technical University of Denmark, Lyngby, Denmark

**Keywords:** Cellular neuroscience, RNA sequencing, Biomedical engineering, Pluripotent stem cells, Biomaterials - cells

Correction to: *Nature Communications* 10.1038/s41467-021-27464-5, published online 15 December 2021.

The original version of this Article contained an error in the Human brain organoid culture section of the Methods, which incorrectly read ‘200 mM L-Ascorbic acid (Sigma-Aldrich, #A4403-100MG)’. The correct version states ‘200 µM L-Ascorbic acid (Sigma-Aldrich, #A4403-100MG)’. This has been corrected in both the PDF and HTML versions of the Article.

