# Supplementary material for: Single-cell transcriptomics captures features of human midbrain development and dopamine neuron diversity in brain organoids
Source: Nat Commun. 2021 Dec 15;12:7302. doi: 10.1038/s41467-021-27464-5 (PMC8674361; doi:10.1038/s41467-021-27464-5)
Supplement: Supplementary file 1 — Supplementary Information [file 41467_2021_27464_MOESM1_ESM.pdf]

Supplementary Information

Fiorenzano et.al., **Single-cell transcriptomics captures features of human midbrain development and dopamine neuron diversity in brain organoids**

Figures 1-9
Supplementary Tables 1,2

Fiorenzano et.al. **Suppl Figure 1**

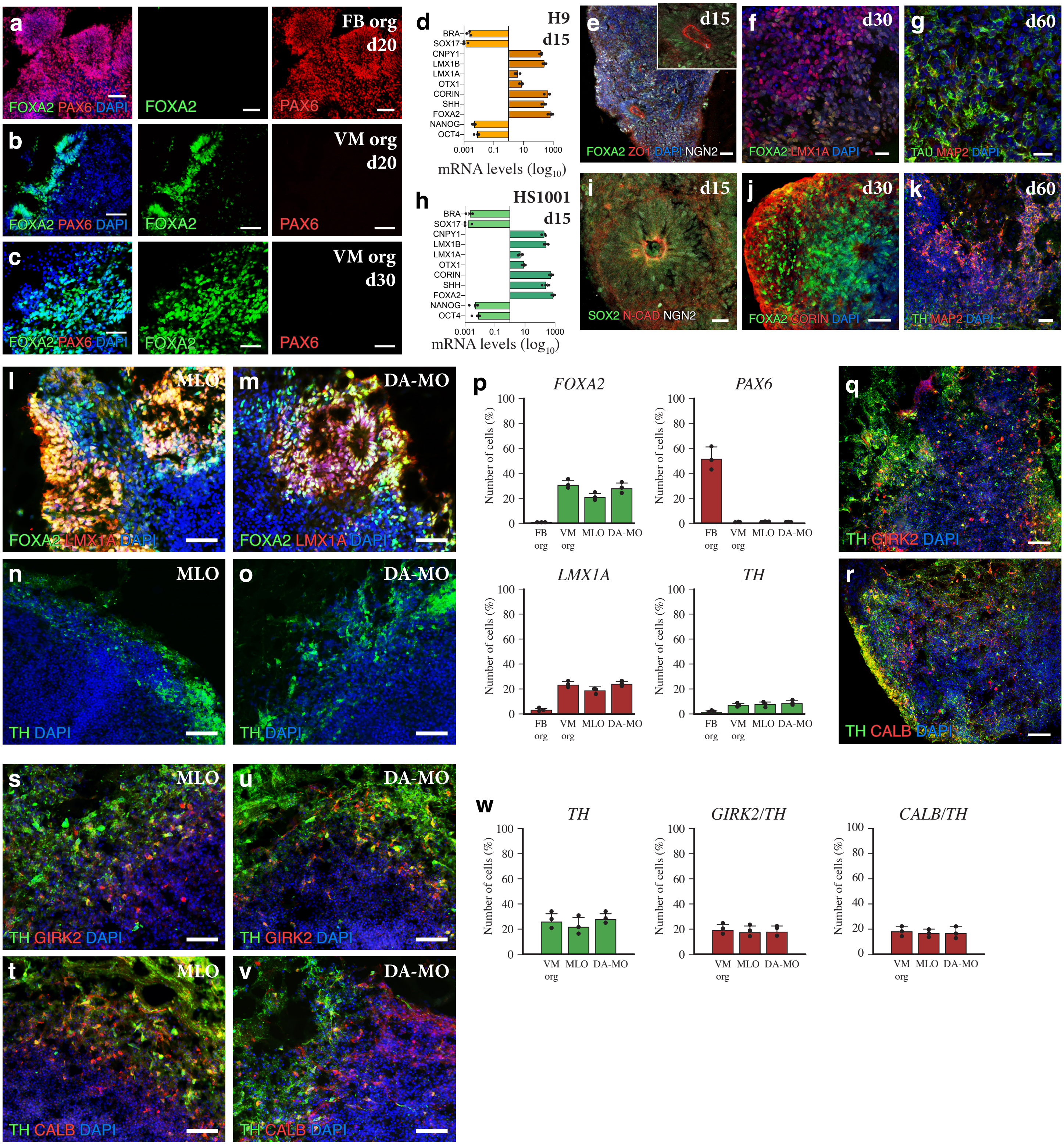

Supplementary Figure 1

a-c, Immunohistochemistry of FOXA2/PAX6 in **a,** forebrain organoids (FBO) at day 20 and **b, c,** in VM organoids across a time course (day 20- 30). Scale bars, 50 μm. **d,** qRT-PCR of selected markers at day 15 during H9-derived VM organoid differentiation. Results are given as fold change over undifferentiated hPSCs. Data represent mean ± SEM obtained from 3 individual organoids. **e,** Immunohistochemistry of FOXA2/ZO-1/NGN2 at day15, **f,** of LMX1A/FOXA2 at day 30, and **g,** of TAU/MAP2 at day 60 during H9 VM organoid differentiation. Scale bars, 50 μm (e) and 20 μm (f,g) **h,** qRT-PCR of selected markers at day 15 during H1001-derived VM organoid differentiation. Results are given as fold change over undifferentiated hPSCs. Data represent mean ± SEM obtained from 3 individual organoids. **i,** Immunohistochemistry of SOXA2/N-CAD at day15, **j,** FOXA2/CORIN at day 30, and **k,** of TH/MAP2 at day 60 during H1001 VM organoid differentiation. Scale bars, 20 μm (i), 50 μm (j) and 100 μm (k). **l, m,** Cryosection of VM organoids at month 1 showing FOXA2/LMX1A double staining in MLOs and DA-MOs. Scale bars, 100 μm. **n, o,** Immunohistochemistry of TH in MLO and DA-MO at month 1. Scale bars, 100 μm. **p,** Percentages of $FOXA2^+$, $PAX6^+$, $LMX1A^+$ and TH^+ cells at month 1 in our VM organoids and in MLOs, DA-MOs and FB organoids. Data represent mean ± SEM of 3 independent organoids. **q-v,** Immunohistochemistry of TH/GIRK2 (q) and TH/CALB (r) at day 60, in our VM organoids and, in **s-v,** MLOs (s,t) and DA-MOs (u,v). Scale bars, 100 μm (q,r) and 50 μm (s-v). **w,** Percentages of TH^+, $GIRK2^+$ and $CALB^+$ a at month 2 in our VM organoid and in MLOs and DA-MOs. Data represent mean ± SEM obtained from 3 individual organoids.

Nuclei were stained with DAPI.

Source data are provided as a Source Data file.

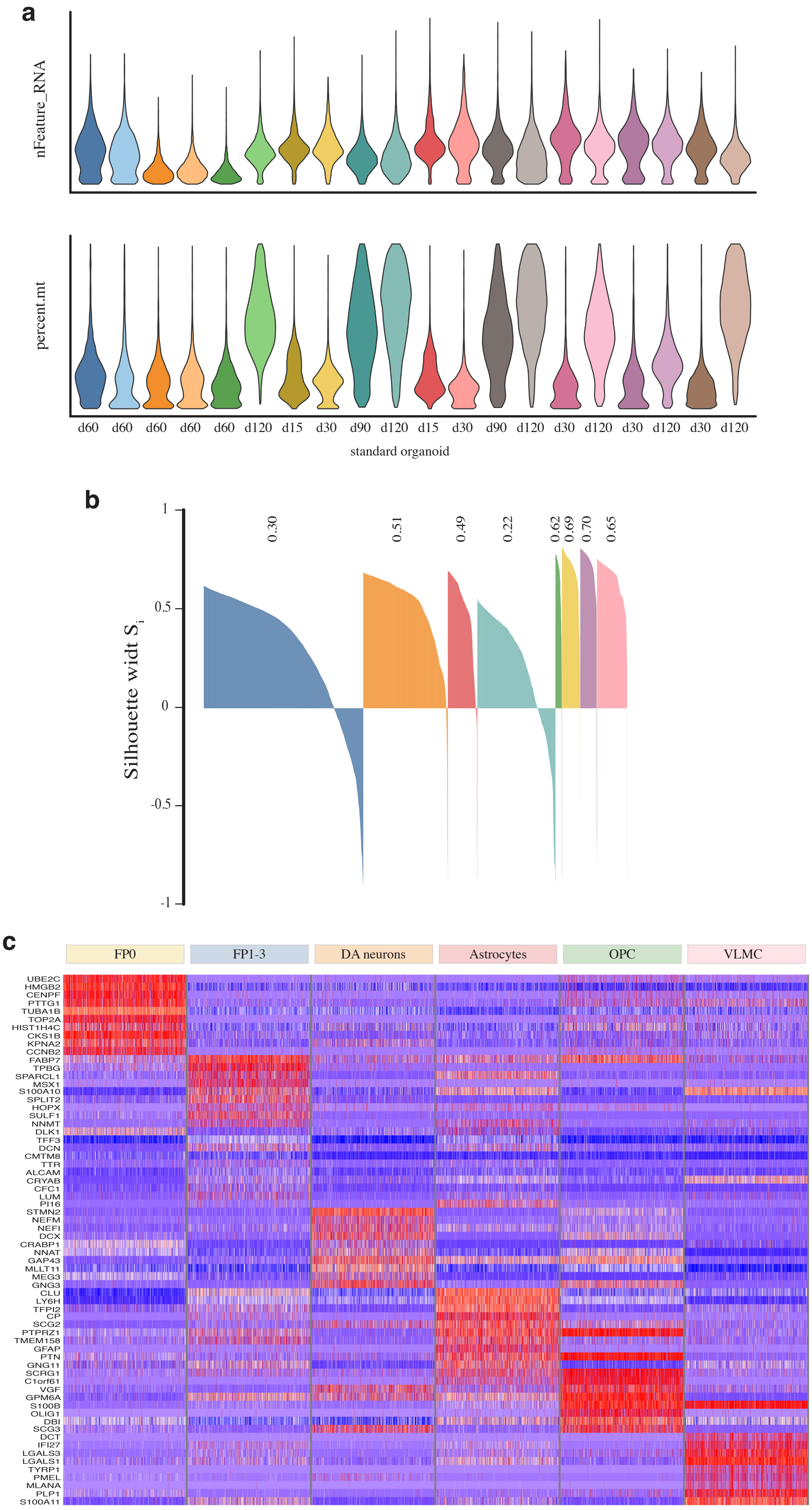
a
nFeature_RNA
percent.mt
d60 d60 d60 d60 d60 d120 d15 d30 d90 d120 d15 d30 d90 d120 d30 d120 d30 d120 d30 d120
standard organoid
b
Silhouette widt S_i
1
0.5
0
-0.5
-1
0.30
0.51
0.49
0.22
0.62
0.69
0.70
0.65
c
FP0
FP1-3
DA neurons
Astrocytes
OPC
VLMC
UBE2C
HMGB2
CENPF
PTTG1
TUBA1B
TOP2A
HIST1H4C
CKS1B
KPNA2
CCNB2
FABP7
TPBG
SPARCL1
MSX1
S100A10
SPLIT2
HOPX
SULF1
NNMT
DLK1
TFF3
DCN
CMTM8
TTR
ALCAM
CRYAB
CFC1
LUM
PI16
STMN2
NEFM
NEFl
DCX
CRABP1
NNAT
GAP43
MLLT11
MEG3
GNG3
CLU
LY6H
TFPI2
CP
SCG2
PTPRZ1
TMEM158
GFAP
PTN
GNG11
SCRG1
C1orf61
VGF
GPM6A
S100B
OLIG1
DBI
SCG3
DCT
IFI27
LGALS3
LGALS1
TYRP1
PMEL
MLANA
PLP1
S100A11

Supplementary Figure 2

a, Filtering of low quality cells based on and number of detected genes per cell and mitochondrial fraction. **b,** Silhouette plot and scores showing the difference between mean distance to cells within the same and nearest clusters. **c,** Heatmap visualizing normalized and scaled expression of top ten enriched genes in VM organoid for each cluster.

Fiorenzano et.al. **Suppl Figure 3**

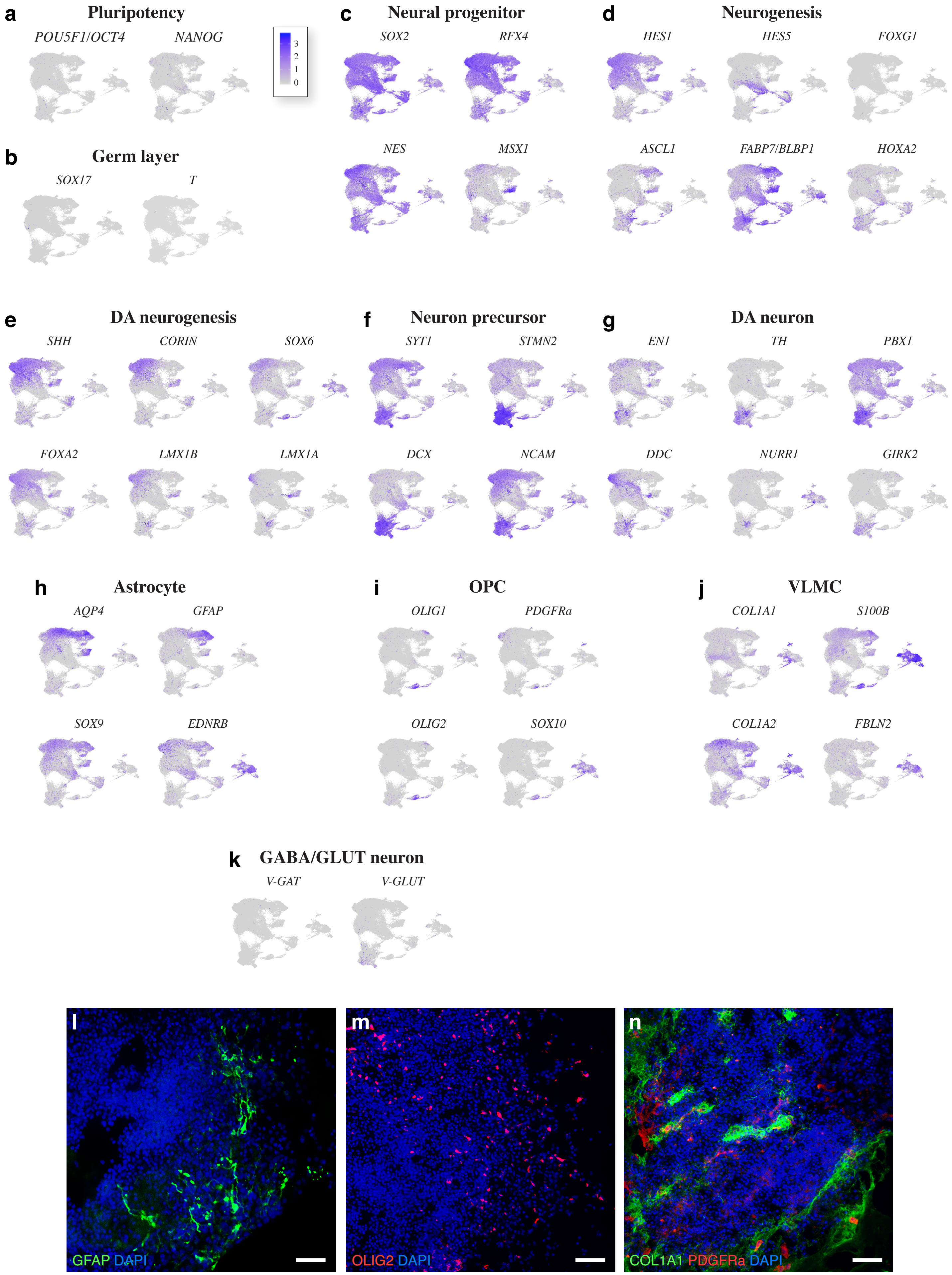

Supplementary Figure 3

a-k, Feature plots visualizing specific gene expression across clusters. Colors indicated expression level. **l-n,** Immunohistochemistry of GFAP, OLIG2 and COL1A1/PDGFRa at day 120 during H9 VM organoid differentiation. Scale bars, 100 μm.

Nuclei were stained with DAPI.

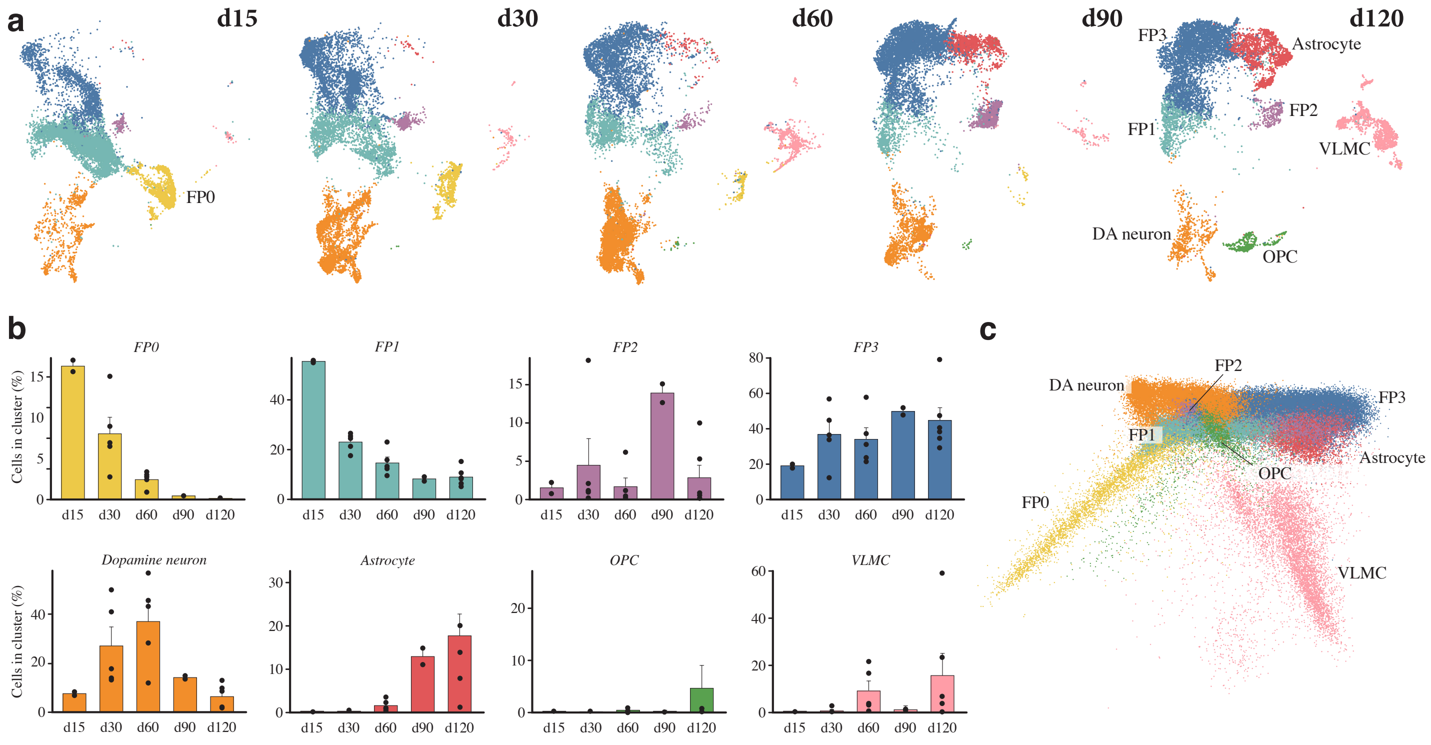

Supplementary Figure 4

a, UMAP plots of cell clusters for each time point (day 15, n=2; day30, n=4; day 60, n=5; day 90, n=2; day 120, n=6). **b,** Fraction of cells from each individual identified cluster from day 15 to day 120. Error bars indicates ± SEM of 2-6 replicates. **c,** Slingshot pseudotime developmental trajectory reconstruction during VM organoid differentiation (day 15–120).

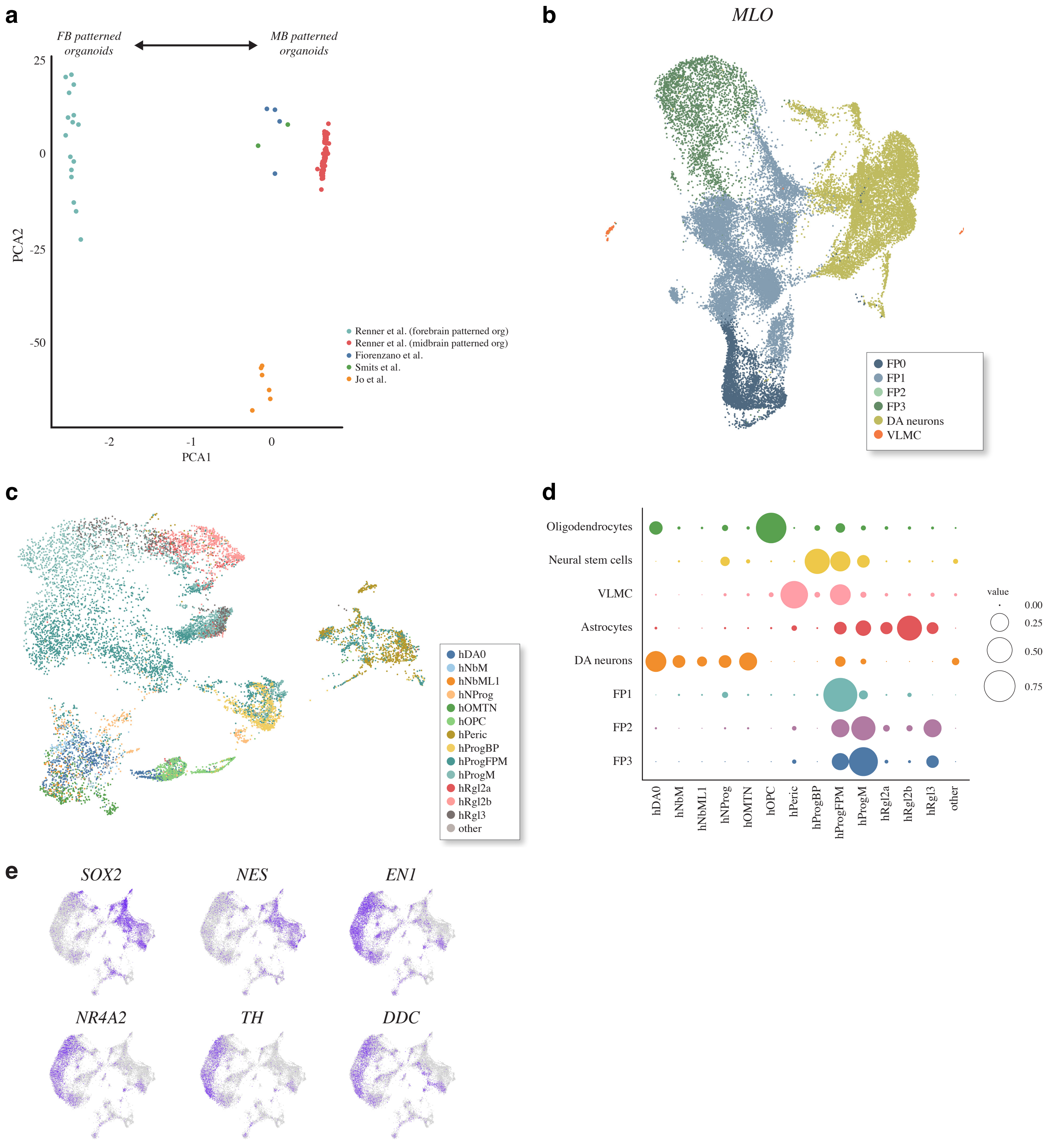

Supplementary Figure 5

a, Principal component (PC) analysis by merging published bulk and scRNAseq dataset from midbrain-patterned organoids from pluripotent stem cells (Jo et al [15]; Fiorenzano et al) and neural progenitors (Renner et al [20]; Smits et al [21]) generated and FBO (Renner et al [16]). **b,** UMAP plot showing clustering of 29,112 cells analyzed cells from MLO organoids at day 30. **c,** UMAP cluster integration analysis combining a published scRNAseq datasets of fetal human midbrain [34] and the hPSC-derived VM organoids with **d,** relative overlapping quantification. **e,** Feature plots visualizing specific gene expression across DA clusters from scRNAseq dataset of human fetal VM and human fetal VM 3D cultures. Colours indicated expression level.

a

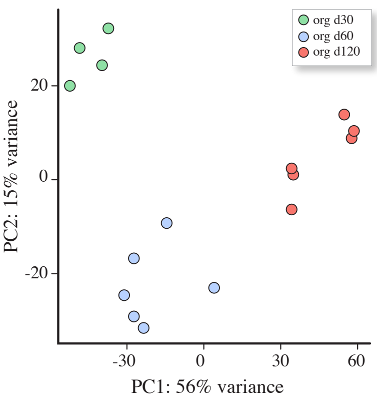

b

Passive property for patched cells
Descriptive statistic with Mean and Std
for each sample

Condition	Membrane capacitance (pF)
organoid external	26.82±15.24
organoid internal	17.11±14.10

VM organoid external
(n=16) number analyzed cells
VM organoid internal
(n=20) number analyzed cells

c

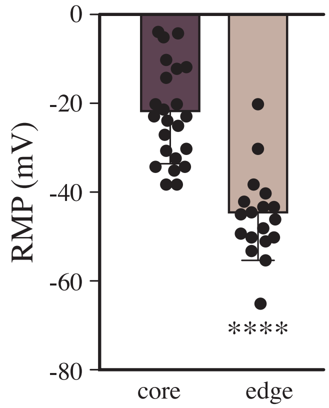

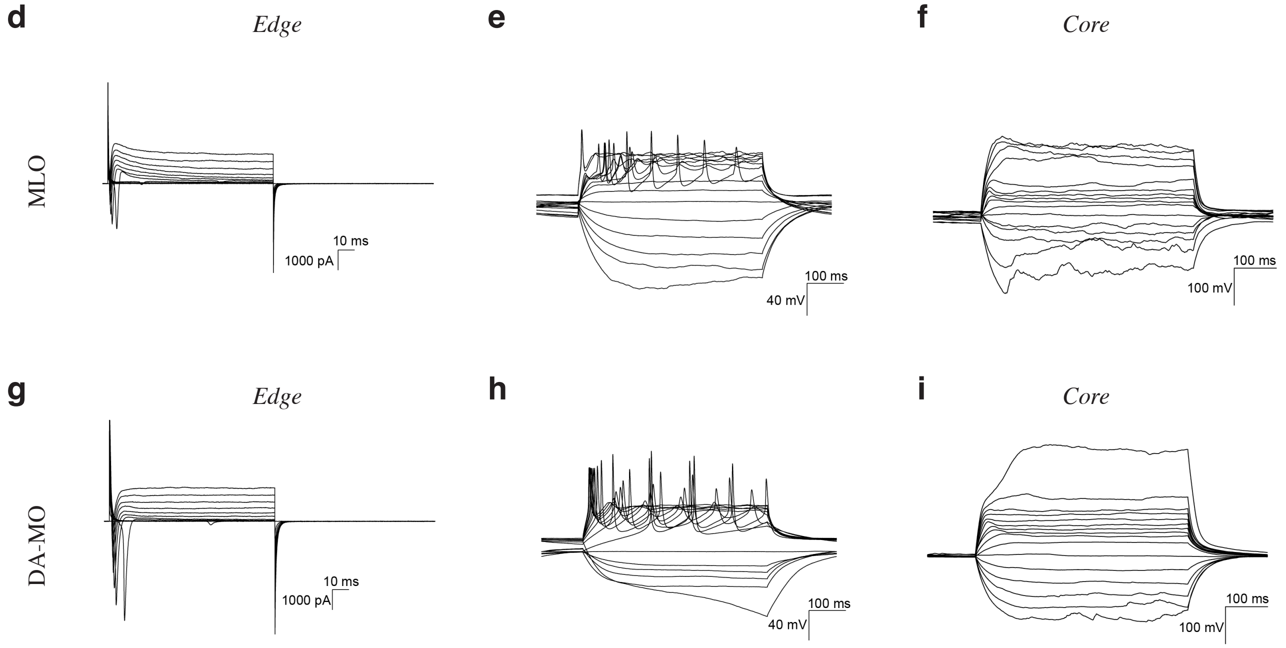

Supplementary Figure 6

a, Principal component (PC) analysis of averaged expression for all cells in VM organoids at day 30, 60, and 120. PC1 explaining 56% variance of organoid groups at different time points; PC2 accounting for 15% variance in biologically independent VM organoids. **b,** Schematic overview reporting passive electrical properties of VM organoid cell membranes from whole-cell patch-clamp recordings. **c,** Resting membrane potential (RMP)quantifications between externally (n=16) and internally (n=20) localized cells in VM organoids at day 90. Silk external (n=18 cells), silk internal (n=23 cells). Data represent mean ± SD. Unpaired two tailed t test, p= 0,0001. **d,** Representative trace from external patching showing inward sodium- and outward potassium-rectifying current traces of MLO at day 70 triggered by stepwise depolarization (n=8). **e,** Patch-clamp recordings of external MLO cells depicting current-induced action potentials (APs) (-85 pA to +165 pA with 20 pA steps) (n=8). **f,** Patch-clamp recordings of internal MLO cells depicting absence of current-induced APs at day 70 (-85 pA to +165 pA with 20 pA steps) (n=3). **g,** Representative trace from external patching showing inward sodium- and outward potassium-rectifying current traces of DA-MO at day 70 triggered by stepwise depolarization (n=3). **h,** Patch-clamp recordings of external DA-MO cells depicting current-induced action potentials (APs) (-85 pA to +165 pA with 20 pA steps) (n=3). **i,** Patch-clamp recordings of internal DA-MO cells depicting absence of current-induced APs at day 90 (-85 pA to +165 pA with 20 pA steps) (n=3).
Source data are provided as a Source Data file.

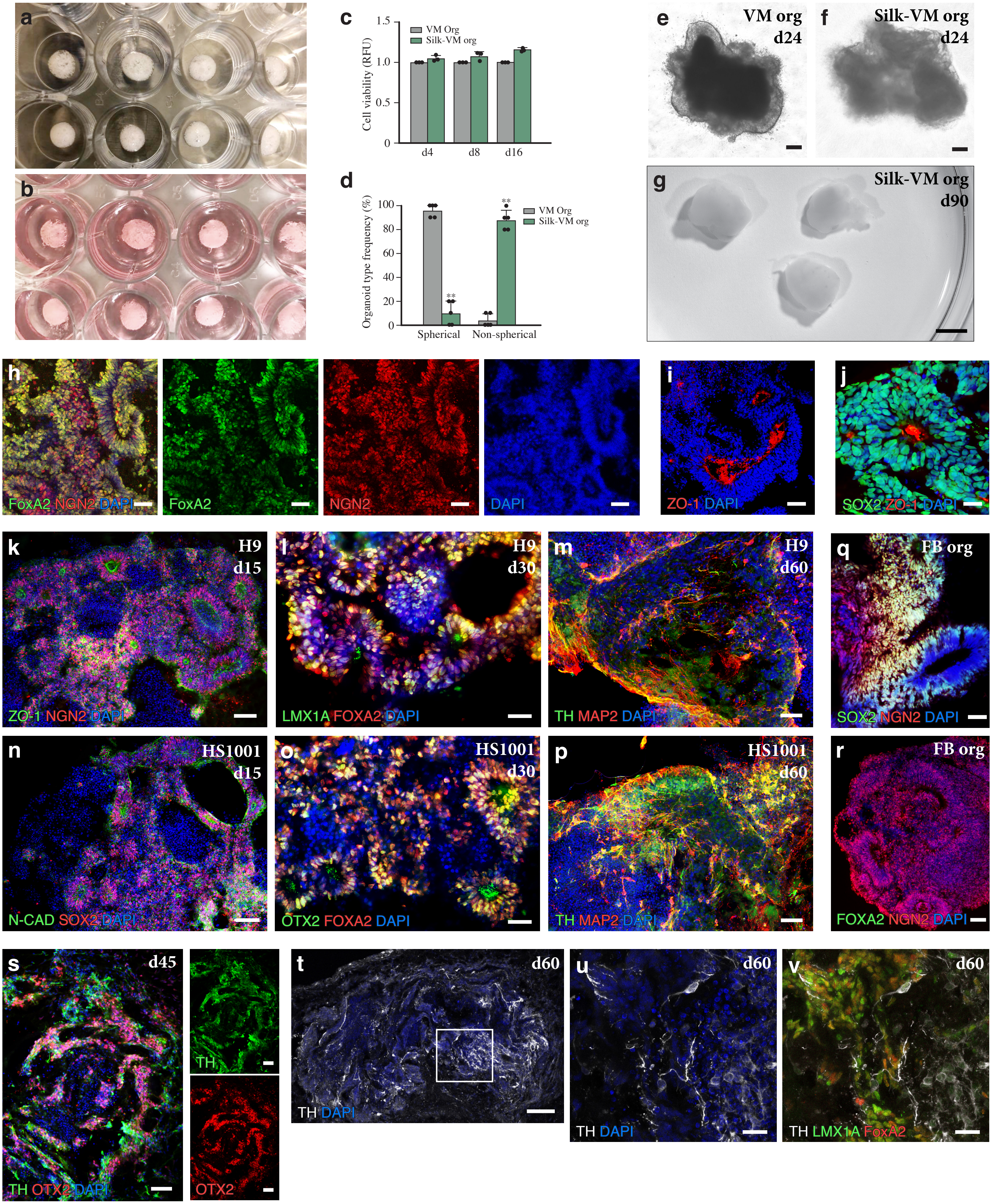
a
b
c
1.5
1.0
0.5
0
Cell viability (RFU)
VM Org
Silk-VM org
d4
d8
d16
d
Organoid type frequency (%)
100
80
60
40
20
0
**
**
Spherical
Non-spherical
e
VM org
d24
f
Silk-VM org
d24
g
Silk-VM org
d90
h
FoxA2 NGN2 DAPI
FoxA2
NGN2
DAPI
i
ZO-1 DAPI
j
SOX2 ZO-1 DAPI
k
H9
d15
ZO-1 NGN2 DAPI
l
H9
d30
LMX1A FOXA2 DAPI
m
H9
d60
TH MAP2 DAPI
q
FB org
SOX2 NGN2 DAPI
n
HS1001
d15
N-CAD SOX2 DAPI
o
HS1001
d30
OTX2 FOXA2 DAPI
p
HS1001
d60
TH MAP2 DAPI
r
FB org
FOXA2 NGN2 DAPI
s
d45
TH OTX2 DAPI
TH
OTX2
t
d60
TH DAPI
u
d60
TH DAPI
v
d60
TH LMX1A FoxA2

Supplementary Figure 7

a, Representative images of silk-foam scaffold before and **b,** after cell dispersion. **c,** Cell viability in VM organoid grown with and without scaffold measured by the CyQuant assay and expressed as relative fluorescence units (RFU). Data represent mean ± SEM obtained from 3 individual organoids per condition. **d,** Organoid-type frequency analysis (spherical vs non-spherical) of VM organoid grown with and without scaffold. Data represent mean ± SD of 5 biologically independent organoids; Mann-Whitney test, p= 0,0079. **e, f,** Representative bright field images of VM organoid grown with and without scaffold at day 24. Scale bars, 100 μm. **g,** Representative bright field images of a long-term silk-VM organoid culture. Scale bar, 1 mm. **h,** Immunohistochemistry of FOXA2/NGN2, **i,** ZO1, and **j,** SOX2/ZO1 from silk-VM organoids at day 21. Scale bars, 50 μm (g) and 20 μm (h,i). **k,** Immunohistochemistry of NGN2/ZO-1 at day 15, **l,** LMX1A/ FOXA2 at day 30, and **m,** TH/MAP2 at day 60 during H9 VM organoid differentiation. Scale bars, 100 μm. **n,** Immunohistochemistry of SOX2/NCAD at day 15, **o,** OTX2/FOXA2 at day 30, and **p,** TH/MAP2 at day 60 during HS1001 VM organoid differentiation. Scale bars, 100 μm. **q,** Immunohistochemistry of SOX2/NGN2 and **r,** FOXA2/NGN2 at day 20 in FBOs. Scale bars, 50 μm(q) and 100 μm(r). **s,** Immunohistochemistry of TH/OTX2 in silk-VM organoid at day 45. Scale bar, 100 μm. **t-v,** Immunohistochemistry of TH/LMX1A/FOXA2 in silk-VM organoid at day 60. Scale bars, 100 μm (t) and 20 μm (u,v). Nuclei were stained with DAPI.

Source data are provided as a Source Data file.

Fiorenzano et.al. **Suppl Figure 8**

a

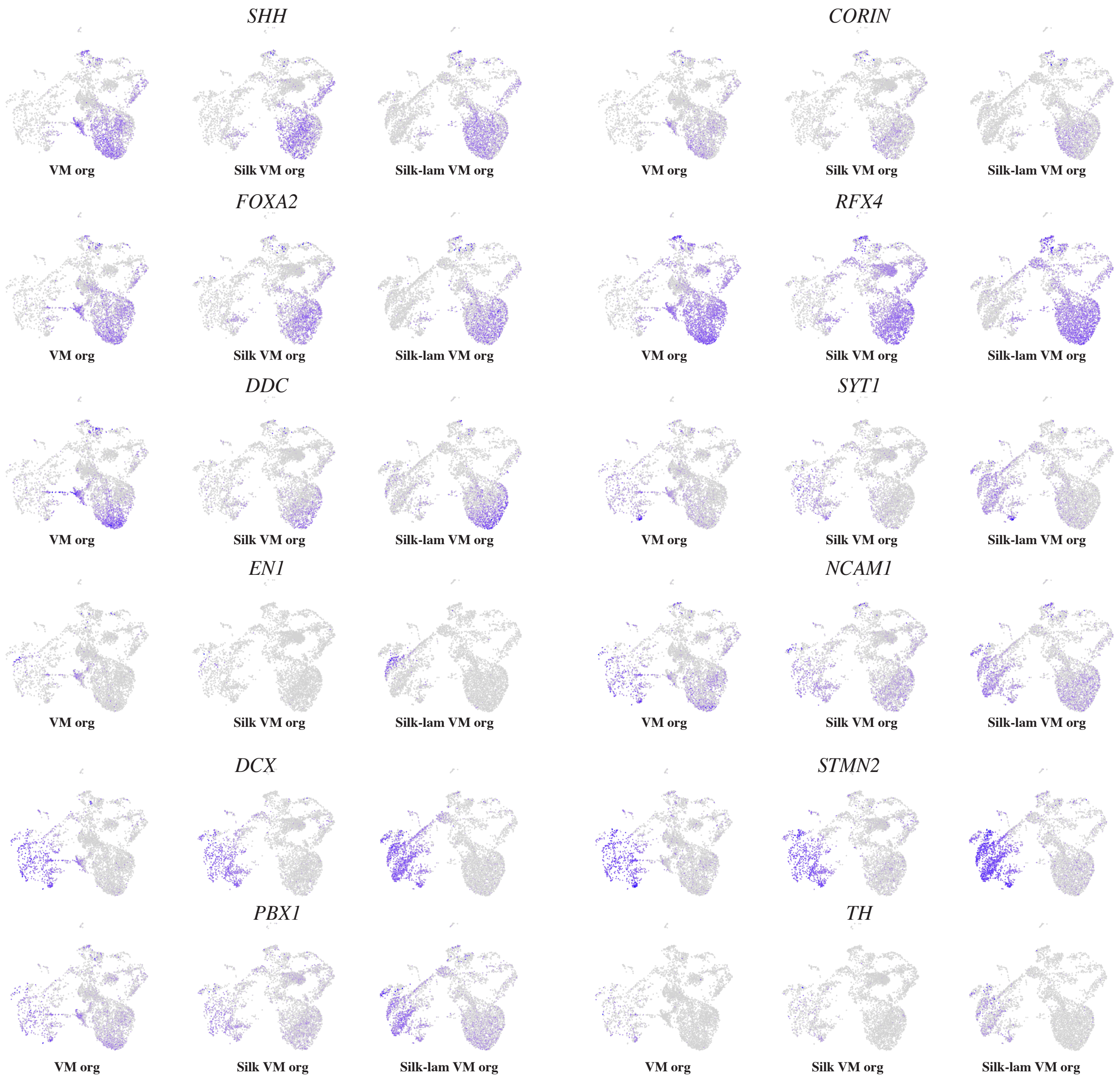

b

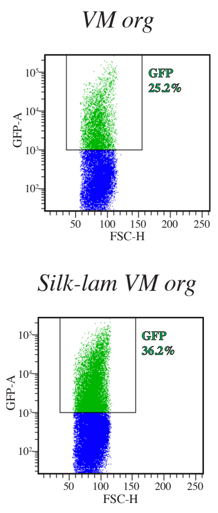

c

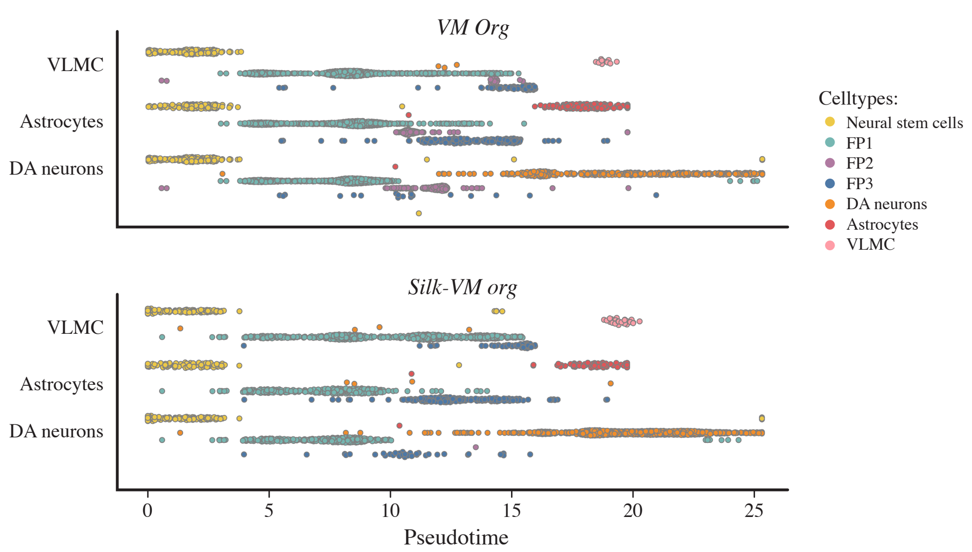

Supplementary Figure 8

a, Feature plots visualizing specific gene expression across identified clusters. Colours indicated expression level. **b,** Representative FACS-plots of GFP expression in conventional and silk-lam VM organoids differentiated from a CRISPR/Cas9-mediated gene-edited *TH*-Cre hPSC line. **c,** Pseudotime ordering of single cells using Slingshot from integrated data of VM and silk-VM organoids from day 30, 60, and 120 (total 5000 cells). Each data point represents a single cell color-coded by cell type. Pseudotimes were calculated by orthogonal projection of Slingshot derived MST.

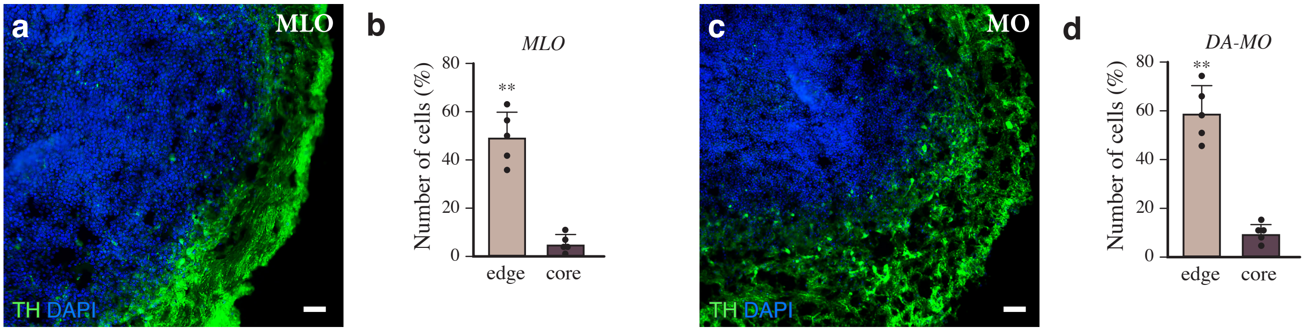

e Passive property for patched cells

Condition	Membrane capacitance (pF)
organoid external	31.77±7.6
organoid internal	29.30±17.02

Silk-lam VM organoid external (n=20) number analyzed cells
Silk-lam VM organoid internal (n=19) number analyzed cells

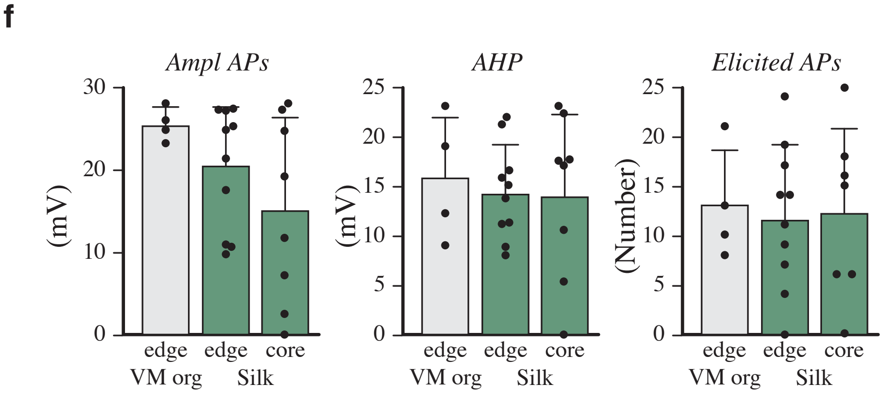

Supplementary Figure 9

a, Immunohistochemistry of TH at month 2 and **b,** its quantification in the outer and inner layers in MLOs. Data represent mean ± SEM obtained from 5 individual organoids; two-tailed Mann-Whitney test, p =0.0079. Scale bar, 100 μm. **c,** Immunohistochemistry of TH at month 2 and **d,** its quantification in the outer and inner layers in in DA-MOs. Data represent mean ± SEM obtained from 5 individual organoids; two-tailed Mann-Whitney test, p =0.0079. Scale bar, 100 μm. **e,** Schematic overview reporting passive electrical properties of silk-lam VM organoid cell membranes from whole-cell patch-clamp recordings. **f,** Amplitude (Ampl), Afterhyperpolarization (AHP) (afterhyperpolarization) and Elicited Action Potentials (APs) analysis of the edge in conventionally generated VM organoids (n= 4) and edge (n=10) and core (n=8) layers in silk-lam VM Organoids. Data represent mean ± SD. Ordinary one-way ANOVA.
Source data are provided as a Source Data file.

Supplementary Table1

Sequence of qPCR primers

GENE NAME	FORWARD PRIMER	REVERSE PRIMER
ACTB	CCTTGCACATGCCGGAG	GCACAGAGCCTCGCCTT
GAPDH	TTGAGGTCAATGAAGGGGTC	GAAGGTGAAGGTCGGAGTCA
CORIN	CATATCTCCATCGCCTCAGTTG	GGCAGGAGTCCATGACTGT
FOXA2	CCGTTCTCCATCAACAACCT	GGGGTAGTGCATCACCTGTT
OTX1	TATAAGGACCAAGCCTCATGGC	TTCTCCTCTTTCATTCCTGGGC
LMX1A	CGCATCGTTTCTTCTCCTCT	CAGACAGACTTGGGGCTCAC
LMX1B	CTTAACCAGCCTCAGCGACT	TCAGGAGGCGAAGTAGGAAC
CNPY1	TTGGCCTCTCAAACACCATTCT	GAGCGAAACAAAACGCAATCAC
EN1	CGTGGCTTACTCCCCATTTA	TCTCGCTGTCTCTCCCTCTC
TH	CGGGCTTCTCGGACCAGGTGTA	CTCCTCGGCGGTGTACTCCACA
BRACHYURY	ATGCAGTGACTTTTTGTCGTGG	ACTGAGGCTGCATTTCCTTCTT
SOX17	CCAGACCGCGACAGGCCAGAAC	AGTGAGGCACTGAGATGCCCCG
OCT4	TCTCCAGGTTGCCTCTCACT	GTGGAGGAAGCTGACAACAA
NANOG	TTGGGACTGGTGGAAGAATC	GATTTGTGGGCCTGAAGAAA

Supplementary Table2

List of antibodies used throughout the study

Antigen	Species	Company cat.no	Dilution
NGN2	Goat	Santa Cruz 19234	1:600
ZO-1	Mouse	Thermo Fisher 1A12	1:300
aPKC	Mouse	Santa Cruz 393219	1:1000
N-CAD	Mouse	BD610920	1:500
KI67	Mouse	BD 550609	1:500
CORIN	Rat	R&D MAB2209	1:200
bIII-Tubulin	Rabbit	Biosite PBR435P	1:1000
MASH1	Mouse	BD 556604	1:200
SOX2	Mouse	R&D MAB2018	1.500
FOXA2	Goat	Santa Cruz (sc-6554)	1:1000
FOXA2	Mouse	Santa Cruz-101060	1:1000
COLIA1	Sheep	R&D 6220	1:1000
LMX1A	Rabbit	Merck Millipore (AB10533)	1:1000
OTX2	Goat	R&D Systems (AF1979)	1:2000
TAU	Rabbit	DAKO A0024	1:1000
TH	Rabbit	Merck Millipore (AB152)	1:1000
TH	Mouse	Immunostar (22941)	1:500
MAP2	Chicken	AbCAM 5392	1.2000
GIRK2	Rabbit	Alamone Labs (APC006	1:500
CALB	Rabbit	Swant cb38	1:500
DDC	Rabbit	Millipore 1519	1:500
DAT	Rabbit	Santa Cruz 14002	1:300
GFAP	Mouse	BioLegend (SMI 21)	1:500
OLIG2	Rabbit	Neuromics RA 25081	1:500
PAX6	Rabbit	Biolegend 901301	1:300
HIF1alpha	Rabbit	GeneTex 127309	1:1000
