# Supplementary material for: Single-cell transcriptomics captures features of human midbrain development and dopamine neuron diversity in brain organoids
Source: Nat Commun. 2021 Dec 15;12:7302. doi: 10.1038/s41467-021-27464-5 (PMC8674361; doi:10.1038/s41467-021-27464-5)
Supplement: Supplementary file 2 — Description of Additional Supplementary Files [file 41467_2021_27464_MOESM2_ESM.docx]

Description of Additional Supplementary Files

Title: Supplementary Movie 1

Description: Immunolabeling-enabled imaging of solvent-cleared organs (iDISCO) reconstructed DA region complexity throughout the entire conventional and silk-engineered brain organoids.
