# Supplementary material for: Single-cell transcriptomics captures features of human midbrain development and dopamine neuron diversity in brain organoids
Source: Nat Commun. 2021 Dec 15;12:7302. doi: 10.1038/s41467-021-27464-5 (PMC8674361; doi:10.1038/s41467-021-27464-5)
Supplement: Supplementary file 4 — Reporting Summary [file 41467_2021_27464_MOESM4_ESM.pdf]

nature portfolio

Corresponding author(s): Malin Parmar, Alessandro Fiorenzano

Last updated by author(s): Oct 29, 2021

Reporting Summary

Nature Portfolio wishes to improve the reproducibility of the work that we publish. This form provides structure for consistency and transparency in reporting. For further information on Nature Portfolio policies, see our Editorial Policies and the Editorial Policy Checklist.

Statistics

For all statistical analyses, confirm that the following items are present in the figure legend, table legend, main text, or Methods section.

n/a	Confirmed	
☐	☒	The exact sample size (n) for each experimental group/condition, given as a discrete number and unit of measurement
☐	☒	A statement on whether measurements were taken from distinct samples or whether the same sample was measured repeatedly
☐	☒	The statistical test(s) used AND whether they are one- or two-sided<br>*Only common tests should be described solely by name; describe more complex techniques in the Methods section.*
☒	☐	A description of all covariates tested
☒	☐	A description of any assumptions or corrections, such as tests of normality and adjustment for multiple comparisons
☐	☒	A full description of the statistical parameters including central tendency (e.g. means) or other basic estimates (e.g. regression coefficient) AND variation (e.g. standard deviation) or associated estimates of uncertainty (e.g. confidence intervals)
☐	☒	For null hypothesis testing, the test statistic (e.g. F, t, r) with confidence intervals, effect sizes, degrees of freedom and P value noted<br>*Give P values as exact values whenever suitable.*
☒	☐	For Bayesian analysis, information on the choice of priors and Markov chain Monte Carlo settings
☒	☐	For hierarchical and complex designs, identification of the appropriate level for tests and full reporting of outcomes
☐	☒	Estimates of effect sizes (e.g. Cohen's d, Pearson's r), indicating how they were calculated

Our web collection on statistics for biologists contains articles on many of the points above.

Software and code

Policy information about availability of computer code

Data collection	The 10x scRNA-seq data generated in this study have been deposited in the Gene Expression Omnibus under accession code GSE168323 (https://www.ncbi.nlm.nih.gov/geo/query/acc.cgi?acc=GSE168323)
Data analysis	Analysis was done using standard scRNA-seq softwares (eg. cellranger v3.0.0, Seurat v3.0.0, R v3.6.1). The code used to generate main figures/ findings together with intermediary files (ie. count matrices) are available in the GitHub-repository. Quantification of fluorescence was performed using with Image J software (NIH, v1.49). FACS plots were generated using FlowJo software. Graphpad Prism (v8) has been used.

For manuscripts utilizing custom algorithms or software that are central to the research but not yet described in published literature, software must be made available to editors and reviewers. We strongly encourage code deposition in a community repository (e.g. GitHub). See the Nature Portfolio guidelines for submitting code & software for further information.

Data

Policy information about availability of data

All manuscripts must include a data availability statement. This statement should provide the following information, where applicable:

- Accession codes, unique identifiers, or web links for publicly available datasets
- A description of any restrictions on data availability
- For clinical datasets or third party data, please ensure that the statement adheres to our policy

The code used for this paper is available on GitHub https://github.com/ParmarLab/scRNA-seq_silk_organoids with DOI: https://doi.org/10.5281/zenodo.5603294

Field-specific reporting

Please select the one below that is the best fit for your research. If you are not sure, read the appropriate sections before making your selection.

- [x] Life sciences
- [ ] Behavioural & social sciences
- [ ] Ecological, evolutionary & environmental sciences

For a reference copy of the document with all sections, see nature.com/documents/nr-reporting-summary-flat.pdf

Life sciences study design

All studies must disclose on these points even when the disclosure is negative.

Sample size	In this study, we aimed to sequence as many single cells from human ventral midbrain organoid at different developmental stages. We achieved 123,294 single cells that allowed robust and reliable data analysis. No statistical methods were used to predetermine sample size.
Data exclusions	No sample was excluded for this study. Single cells were filtered using standard criteria as detailed in the manuscript (ie. on number of detected genes, % mitochondrial fraction).
Replication	All experiment including scRNA seq, RT-PCR, IFs as well as functional assessment have been validated at least in 3 separate experiments. Particularly, VM organoid differentiation using conventional and silk methodology (Fig.1,5) have been tested using 3 different PSC lines. 16 independent VM organoids from 4 biologically replicates were analyzed for FACS analysis in Fig 6i.
Randomization	Randomization of samples were not applicable in this paper.
Blinding	The investigators who performed immunostainings quantification, RNA-seq analysis and electrophysiology were blinded to the methodologies used for brain organoid generation.

Reporting for specific materials, systems and methods

We require information from authors about some types of materials, experimental systems and methods used in many studies. Here, indicate whether each material, system or method listed is relevant to your study. If you are not sure if a list item applies to your research, read the appropriate section before selecting a response.

Materials & experimental systems

n/a	Involved in the study
[ ]	[x] Antibodies
[ ]	[x] Eukaryotic cell lines
[x]	[ ] Palaeontology and archaeology
[x]	[ ] Animals and other organisms
[x]	[ ] Human research participants
[x]	[ ] Clinical data
[x]	[ ] Dual use research of concern

Methods

n/a	Involved in the study
[x]	[ ] ChIP-seq
[ ]	[x] Flow cytometry
[x]	[ ] MRI-based neuroimaging

Antibodies

Antibodies used

anti-NGN2 (Goat), 1:600, Santa Cruz 19234
anti-ZO-1 (Mouse), 1:300, Thermo Fisher 1A12
anti-aPKC (Mouse), 1:1000, Santa Cruz 393219
anti-N-CAD (Mouse), 1:500, BD610920
anti-KI67 (Mouse), 1:500, BD 550609
anti-CORIN (Rat), 1:200, R&D MAB2209
anti-bIII-Tubulin (Rabbit), 1:1000, Biosite PBR435P
anti-MASH1 (Mouse), 1:200, BD 556604
anti-SOX2 (Mouse), 1:500, R&D MAB2018
anti-FOXA2 (Goat), 1:1000, Santa Cruz (sc-6554)
anti-FOXA2 (Mouse), 1:1000, Santa Cruz-101060
anti-COLIA1 (Sheep), 1:1000, R&D 6220
anti-LMX1A (Rabbit), 1:1000, Merck Millipore (AB10533)
anti-OTX2 (Goat), 1:2000, R&D Systems (AF1979)
anti-TAU (Rabbit), 1:1000, DAKO A0024
anti-TH (Rabbit), 1:1000, Merck Millipore (AB152)
anti-TH (Mouse), 1:500, Immunostar (22941)
anti-MAP2 (Chicken), 1.2000, AbCAM 5392

anti-GIRK2 (Rabbit), 1:500, Alamone Labs (APC006)
anti-CALB (Rabbit), 1:500, Swant cb38
anti-DDC (Rabbit), 1:500, Millipore 1519
anti-DAT, (Rabbit), 1:300, Santa Cruz 14002
anti-GFAP, (Mouse),1:500, BioLegend (SMI 21)
anti-OLIG2, (Rabbit) 1:500, Neuromics RA 25081
anti-PAX6 (Rabbit), 1:300, Biolegend 901301
anti-HIF1alpha (Rabbit), 1:1000, GeneTex 127309

Validation	The antibodies used in this work have been used in previously published reports and/or validated from the companies where we purchased the antibodies

Eukaryotic cell lines

Policy information about cell lines

Cell line source(s)	RC17: hPSCreg RCe021-A obtained from Roslin Cells<br>H9: hPSCreg WAe009-A obtained from WiCell<br>HS999 and HS1001 obtained from Outi Hovatta, Karolinska Institute<br>The study includes human fetal tissue. It was collected from a legally terminated embryo collected in accordance with existing guidelines with approval of the Swedish National Board of Health and Welfare and informed consent from women seeking elective abortions.
Authentication	PSC lines were authenticated through immunostaining against several pluripotency markers including OCT4, NANOG and SOX2
Mycoplasma contamination	all cell lines tested negative for mycoplasma comtamination
Commonly misidentified lines (See ICLAC register)	No commonly misidentified lines were used

Flow Cytometry

Plots

Confirm that:

- [x] The axis labels state the marker and fluorochrome used (e.g. CD4-FITC).
- [x] The axis scales are clearly visible. Include numbers along axes only for bottom left plot of group (a 'group' is an analysis of identical markers).
- [x] All plots are contour plots with outliers or pseudocolor plots.
- [x] A numerical value for number of cells or percentage (with statistics) is provided.

Methodology

Sample preparation	hVM organoid differentiated from hPSCs, were dissociated into a single cell suspension using the papain kit (Worthington). Human brain organoids were collected in 2ml tube with papaine and incubate for at 37°C on the wheel . The cloudy cell suspension were resuspend in 1ml of cold D-PBS+0,01%BSA filter through 20um mesh.
Instrument	BD FACSAria III Cell Sorter
Software	FlowJo v10.4.2
Cell population abundance	Purity of samples is assessed from live GFP detection
Gating strategy	Cells were analyzed using forward scatter area/side scatter area (FSC-A/SS-A), side scatter width/side scatter area (SSC-W/SSC-A), forward scatter area/forward scatter width (FSC-A/ FSC-W) followed by detection of fluorescent events FITC-A/PE-A. GFP was excited with a 488nm laser.

- [x] Tick this box to confirm that a figure exemplifying the gating strategy is provided in the Supplementary Information.
